# CK2α-mediated phosphorylation of GRP94 facilitates the metastatic cascade in triple-negative breast cancer

**DOI:** 10.1038/s41420-024-01956-x

**Published:** 2024-04-22

**Authors:** Hye-Youn Kim, Young-Mi Kim, Suntaek Hong

**Affiliations:** Department of Biochemistry, Lee Gil Ya Cancer and Diabetes Institute, Gachon University College of Medicine, Incheon, 21999 Republic of Korea

**Keywords:** Breast cancer, Metastasis, Predictive markers

## Abstract

Distant metastasis is a significant hallmark affecting to the high death rate of patients with triple-negative breast cancer (TNBC). Thus, it is crucial to identify and develop new therapeutic strategies to hinder cancer metastasis. While emerging studies have hinted a pivotal role of glucose-regulated protein 94 (GRP94) in tumorigenesis, the exact biological functions and molecular mechanisms of GRP94 in modulating cancer metastasis remain to be elucidated. Our study demonstrated an increased expression of GRP94 in TNBC correlated with metastatic progression and unfavorable prognosis in patients. Functionally, we identified that GRP94 depletion significantly diminished TNBC tumorigenesis and subsequent lung metastasis. In contrast, GRP94 overexpression exacerbated the invasiveness, migration, and lung metastasis of non-TNBC cells. Mechanistically, we found that casein kinase 2 alpha (CK2α) active in advanced breast cancer phosphorylated GRP94 at a conserved serine 306 (S306) residue. This phosphorylation increased the stability of GRP94 and enhanced its interaction with LRP6, leading to activation of canonical Wnt signaling. From a therapeutic standpoint, we found that benzamidine, a novel CK2α inhibitor, effectively suppressed GRP94 phosphorylation, LRP6 stabilization, and metastasis of TNBC. Our results point to the critical role of CK2α-mediated GRP94 phosphorylation in TNBC metastasis through activation of Wnt signaling, highlighting GRP94 as a therapeutic target to impede TNBC metastasis.

## Introduction

Triple-negative breast cancer (TNBC) is a malignant and highly metastatic subtype of breast cancer that lacks expression of estrogen receptor (ER), progesterone receptor (PR), and human epidermal growth factor receptor 2 (HER2) [1]. It accounts for ~15–20% of all breast cancer cases. TNBC is associated with poor survival rate due to limited treatment options and increased likelihood of metastasis [2]. Despite the improvement of therapeutic strategies, the survival rate of breast cancer patients, especially TNBC, remains poor. TNBC has the worst prognosis and distant-free survival due to metastasis [3]. Currently, there are various challenges for TNBC treatment. For example, a large portion of TNBC patients suffers the recurrence after adjuvant therapy [4, 5]. Although it is plausible to develop therapies for hormone receptor and HER2-positive breast cancers, little progress has been made in developing effective druggable targets in TNBC [6]. Consequently, there is an urgent need to identify novel biomarkers that can serve as potential therapeutic targets for TNBC metastasis. The discovery of such biomarkers not only enhances our understanding of the underlying molecular mechanisms driving metastasis, but also holds great promise for the development of targeted therapies. By specifically targeting the molecular pathways associated with metastasis, we can potentially improve patient outcomes and survival rates.

The metastatic cascade is a multi-step process that begins with local invasion of the neighboring extracellular matrix and dissemination to distant organ sites by malignant cells [7, 8]. Epithelial-to-mesenchymal transition (EMT) is a critical step that involves the acquisition of a mesenchymal phenotype and fibroblast-like morphology after loss of epithelial polarity with cytoskeletal reorganization known as invadopodia and metastatic abilities, which allow cancer cells to invade adjacent tissues and spread to distant sites [9–11]. Invadopodia have been studied in a variety of different cell types in the tumor microenvironment. There is also substantial evidence from in vivo studies that support their invasive behavior in the dissemination of tumor cells [12, 13]. In response to various stimuli such as growth factors, hypoxia, and EMT-inducing signals, many structural and regulatory proteins play an important role in the regulation of cellular actin dynamics and dissemination of cancer cells [14, 15]. Despite a widespread characterization of components for invadopodia formation, detailed molecular mechanisms for their regulation are not fully understood yet.

Glucose-regulated protein 94 (GRP94), also known as endoplasmic reticulum (ER) stress protein 94, is a member of the heat shock protein 90 (HSP90) family. It is primarily localized within the ER [16, 17]. It binds to and hydrolyzes ATP [18, 19]. GRP94 is known to regulate many cellular functions, such as protein folding and assembly, ER translocation, ER-associated degradation, and the unfolded protein response pathway [20]. Furthermore, recent studies have shown that GRP94 plays an important role in tumorigenesis of multiple types of cancer, including breast [21, 22], colon [23, 24], liver [25], and lung cancers [26, 27]. Furthermore, GRP94 can interact with key molecular players such as receptor tyrosine kinases and transcription factors in these pathways, thereby modulating their activity and contributing to the aggressive behavior of cancer cells. However, the precise mechanisms by which GRP94 promotes metastasis in TNBC and its specific downstream effectors remain largely unknown.

Emerging evidence suggests that dysregulation of protein kinases plays a critical role in the acquisition of metastatic properties by cancer cells [28, 29]. Among these kinases, Casein kinase 2 (CK2) is a conserved serine/threonine kinase in many species and has recently gained attention due to its involvement in many cellular processes, such as cell growth, death, and migration. CK2 is known to regulate diverse signaling pathways by phosphorylating a wide range of protein substrates involved in cell adhesion, cytoskeletal organization, and EMT [30, 31]. CK2-mediated phosphorylation of key substrates has been implicated as a critical step in metastasis by modulating activities of transcription factors, cell adhesion molecules, and components of signaling pathways. Notably, dysregulation of CK2 expression and activity has been observed in TNBC, indicating its potential involvement in the metastatic progression of this aggressive breast cancer subtype [32, 33].

In this study, we explored deeper mechanisms of how GRP94 could regulate invasion and metastasis of TNBC cells. We first demonstrated that GRP94 was associated with metastatic breast cancer invasion and metastasis using in vitro and in vivo models. We then investigated the role of GRP94 in regulating EMT and invadopodia formation. We also confirmed the importance of post-translational regulation of GRP94 by CK2α for metastatic potential of TNBC cells. Finally, we proposed a specific mechanism for the CK2α-GRP94-LRP6 axis in the invasion and metastasis of TNBC, which might be a potential target to combat metastatic breast cancer.

## Results

### Elevated GRP94 expression correlates with metastatic progression and adverse clinical outcomes in breast cancer

**Fig. 1:**
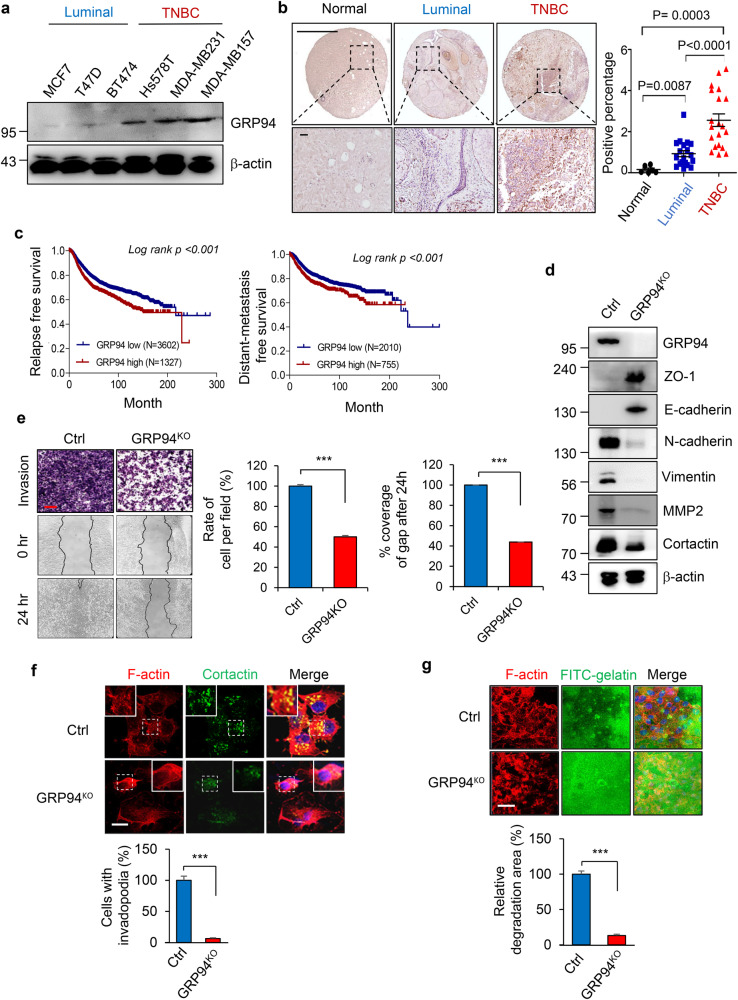
Overexpression of GRP94 is associated with metastatic progression of TNBC cells. **a** Representative western blotting showing GRP94 expression in a panel of luminal and TNBC cell lines. As a normalization control, β-actin band was used. **b** Representative immunohistochemical staining of GRP94 in normal tissues (9 samples), primary breast cancer tissues (40 samples), and metastases (10 samples). Quantification of staining intensity was performed using ImageJ software from at least three fields (right panel). Scale bar = 1000 μm (upper panel), 50 μm (bottom panel). **c** Kaplan–Meier survival analysis of recurrence-free survival (RFS) and distant metastasis-free survival (DMFS) of breast cancer patients. Patients with tumors expressing GRP94 levels above the mean value are shown in red, while those with tumors expressing GRP94 below the mean are shown in blue. Log-rank tests were used for statistical analysis. **d** Validation of epithelial-mesenchymal transition markers (ZO-1, N-cadherin, and Vimentin), metastasis marker (MMP2), and invadopodia marker (Cortactin) in control or GRP94^KO^ MDA-MB231 cells was performed using western blotting. As a normalization control, β-actin band was used. **e** Transwell and wound healing assays were performed for parental and GRP94^KO^ MDA-MB231 cells. The histogram shows average number of migrated and invaded cells per view. Scale bar = 200 μm. ***p < 0.001 (Student’s *t* test). **f** Representative immunofluorescence images showing invadopodia in control and GRP94^KO^ MDA-MB231 cells revealed by F-actin and Cortactin double staining. Inset shows a magnified image of invadopodia. Quantification of the percentage of cells with invadopodia was performed using ImageJ software (bottom panel). ***p < 0.001 (Student’s *t* test). Scale bar = 50 μm. **g** Measurement of invasion activities by indicated cells plated onto FITC-gelatin coated slides based on the intensity of gelatin degradation. The intensity of gelatin degradation was measured using ImageJ software and compared with control cells (bottom panel). ***p < 0.001 (Student’s *t* test). Scale bar = 50 μm. Results are representative of at least three independent experiments and data are presented as mean ± SD.

To investigate the association between GRP94 expression and the metastatic potential of breast cancer, we conducted western blot analysis to assess endogenous GRP94 levels in a panel of breast cancer cell lines, including non-metastatic luminal cell lines (MCF7, T47D, and BT474) and metastatic triple-negative breast cancer (TNBC) cell lines (Hs578T, MDA-MB231 and MDA-MB157). As depicted in Fig. 1a, TNBC cell lines exhibited significantly higher levels of GRP94 expression than luminal cell lines. However, analysis of 52 breast cancer cell lines using the GEO dataset (GSE41313) did not reveal any significant correlation in GRP94 mRNA levels between TNBC and luminal cells (Supplementary Fig. S1a). Subsequently, we performed immunohistochemistry for 45 paraffin-embedded human breast tissue samples, including normal breast tissue (*n* = 6), luminal breast carcinoma (*n* = 19), and TNBC breast carcinoma (*n* = 20) samples to examine GRP94 expression. Results showed a remarkable elevation of GRP94 expression in TNBC tissues compared to GRP94 expression levels in luminal and normal tissues (Fig. 1b). These results suggest that GRP94 expression is increased in TNBC at the post-transcriptional level. Furthermore, Kaplan-Meier survival analysis demonstrated significant associations of positive GRP94 expression with poor relapse-free survival (RFS) and distant-metastasis-free survival (DMSF) outcomes in breast cancer patients (*p* < 0.001; Fig. 1c).

To understand the potential role of GRP94 in breast cancer metastasis, we ectopically overexpressed or depleted GRP94 expression in MCF7 and MDA-MB231 cells, respectively. Western blot analysis revealed that knockout of GRP94 in MDA-MB231 cells resulted in increased expression of epithelial phenotype markers including ZO-1 and E-cadherin with decreased expression of mesenchymal phenotype markers including N-cadherin, Vimentin, MMP2, and Cortactin (Fig. 1d). Interestingly, the restoration of GRP94 in GRP94 knockout MDA-MB231 cells lead to the rescues of expression of EMT markers and metastatic potential (Supplementary Fig. S1b, c). Conversely, overexpression of GRP94 in MCF7 cells attenuated the expression of epithelial phenotype markers but accelerated the expression of a mesenchymal phenotype marker (Supplementary Fig. S1d). Moreover, transwell invasion and wound scratch assays demonstrated weakened invasion and migration abilities of GRP94 knockout MDA-MB231 cells (Fig. 1e), whereas overexpression of GRP94 enhanced the metastatic potential of MCF7 cells (Supplementary Fig. S1e). Furthermore, GRP94 expression exhibited significant correlations with invadopodia formation (Fig. 1f) and gelatin degradation abilities (Fig. 1g and Supplementary Fig. S1d). Collectively, these findings underscore the critical role of GRP94 as a modulator in promoting EMT, invadopodia formation, and invasion of breast cancer cells.

### GRP94 promotes Wnt signaling activation by enhancing stability of LRP6

**Fig. 2:**
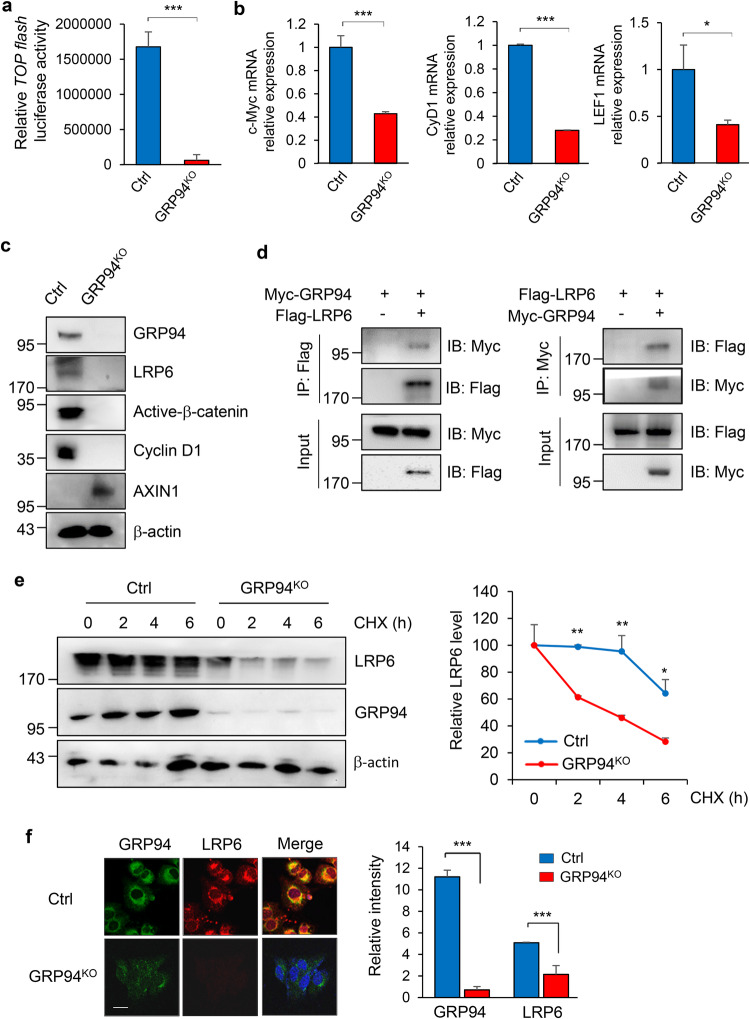
GRP94 regulates LRP6 maturation and WNT pathway signaling. **a** Control or GRP94^KO^ MDA-MB231 cells were transfected with TOPflash/FOPflash reporter plasmids. At 24 h after transfection, β-catenin transcriptional activity was assessed by measuring luminescence. TOPflash values are presented as fold change compared to control cells. As a normalization control, β-galactosidase activity was used. Bars represent mean ± SD. ***p < 0.001 (Student’s *t* test). **b** mRNA expression levels of Wnt target genes in control or GRP94^KO^ MDA-MB231 cells were validated using qRT-PCR. Cyclophilin was used as a normalization control. Statistical significance was calculated using Student’s *t* test. *p < 0.05; ***p < 0.001 (Student’s *t* test). **c** Wnt signaling-related markers were detected in control or GRP94^KO^ MDA-MB231 cells with western blotting. The β-actin band was used as a normalization control. **d** Interaction of GRP94 with LRP6 was confirmed in HEK293 cells by transfecting cells with both Myc-GRP94 and Flag-LRP6 constructs. Immunoprecipitates were detected using indicated antibodies. As input control, 10% of cell lysates were used. **e** Stability of LRP6 was assessed in control or GRP94^KO^ MDA-MB231 cells after treatment with CHX (10 μM) for indicated time (left panel). Relative protein levels of LRP6 normalized to β-actin were plotted as bar graph (right panel). Statistical significance was determined using Student’s *t* test (*p < 0.05; **p < 0.01). The experiment was performed in triplicate. Results are presented as mean ± SD of three independent experiments. **f** Confocal immunofluorescence analysis was performed to check co-localization of GRP94 and LRP6 in control or GRP94^KO^ MDA-MB231 cells. Signal intensity corresponding to GRP94 and LRP6 was quantified using ImageJ software (right panel). Statistical analysis was performed using Student’s *t* test (***p < 0.001). Scale bar = 50 μm. Results are representative of at least three independent experiments and data are presented as mean ± SD.

To gain further insights into the underlying mechanism by which GRP94 promotes metastasis in breast cancer, we focused on the Wnt signaling pathway, considering that GRP94 is a crucial HSP localized in the ER and a critical chaperone for low-density lipoprotein receptor-related protein 6 (LRP6), a key co-receptor involved in Wnt signaling [24]. To explore whether GRP94 could regulate Wnt signaling activity, we conducted a TOPflash luciferase reporter assay, which detects the β-catenin-dependent activation of luciferase construct containing TCF-binding sites. The relative transactivation of TCF reporter was markedly reduced in GRP94-knockouted MDA-MB231 cells compared to that in control cells (Fig. 2a). Consistent with these findings, mRNA and protein levels of Wnt signaling-related markers, c-Myc, Cyclin D1 and LEF1 were decreased upon ablation of GRP94 in MDA-MB231 cells, whereas the expression of AXIN1, a known inhibitor of Wnt signaling, was increased (Fig. 2b, c). Conversely, mRNA and protein levels of Wnt signaling-related markers were elevated in GRP94-overexpressing MCF7 cells accompanied by a decreased AXIN1 expression (Supplementary Fig. S2a, b). Furthermore, immunofluorescence staining revealed weakened fluorescence intensities of β-catenin and Myc in GRP94-knockouted cells (Supplementary Fig. S2c).

To examine whether GRP94 could regulate the Wnt signaling pathway through LRP6, co-immunoprecipitation experiments were conducted. Results confirmed the interaction between GRP94 and LRP6 (Fig. 2d). Subsequently, we investigated whether GRP94 could enhance the stability of LRP6 protein. CHX, a protein synthesis inhibitor, was utilized to assess the effect of GRP94 on LRP6 stability. GRP94-knockouted or parental MDA-MB231 cells were treated with CHX (10 μM) for different durations to inhibit protein synthesis. Degradation rates of existing LRP6 protein were then determined by western blot analysis. Results demonstrated that GRP94 knockout promoted LRP6 degradation compared to the control (Fig. 2e). Additionally, immunofluorescence staining revealed reduced LRP6 expression and co-localization with GRP94 upon GRP94 depletion (Fig. 2f). Collectively, our findings suggest that GRP94 can activate the Wnt signaling pathway by enhancing the stability of LRP6 protein.

### CK2α-mediated phosphorylation of GRP94 facilitates interaction with LRP6 and enhances stabilization

**Fig. 3:**
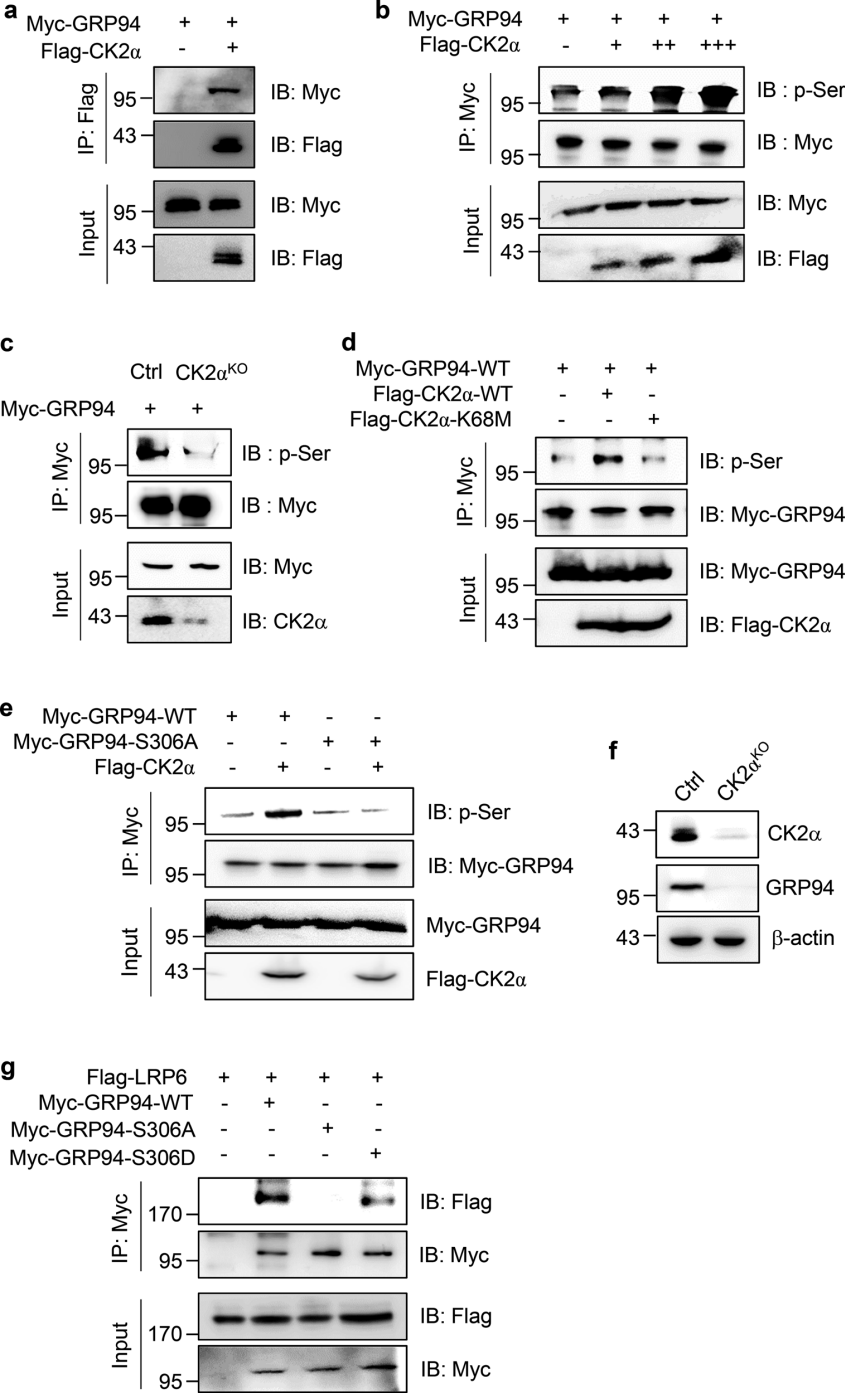
CK2α-mediated GRP94 phosphorylation is required for GRP94 protein stabilization and binding with LRP6. **a** Interaction between GRP94 and CK2α was checked after transfecting HEK293 cells with both Myc-GRP94 and Flag-CK2α constructs. Immunoprecipitation was performed with anti-Flag antibody. Immunoprecipitates were detected using indicated antibodies. For analysis, 10% of cell lysate was used as input. **b** For analysis of CK2α-mediated phosphorylation of GRP94, GRP94 DNA and increasing concentrations of CK2α constructs were used to transfect HEK293 cells. Immunoprecipitation was performed with anti-Myc antibody and immunoprecipitates were detected with anti-p-Ser antibody. **c** To confirm the involvement of CK2α in GRP94 phosphorylation, GRP94 construct was used to transfect CK2α^KO^ cells. GRP94 was then immunoprecipitated with anti-Myc antibody. Immunoprecipitates were analyzed with anti-p-Ser antibody. **d** To validate the effect of kinase activity of CK2α on GRP94 phosphorylation, Myc-GRP94, and CK2α^WT^ or ^K68M^ constructs were used to co-transfect HEK293 cells. Immunoprecipitation was performed with anti-Myc antibody and immunoprecipitates were detected with anti-p-Ser antibody. **e** To confirm the S306 residue as phosphorylation site by CK2α, Myc-GRP94^WT^ or ^S306A^ mutant constructs were used to transfect HEK293 cells. Immunoprecipitation was performed with anti-Myc antibody and immunoprecipitates were detected with anti-p-Ser antibody. **f** Endogenous CK2α and GRP94 protein levels were analyzed in control or CK2α^KO^ MDA-MB231 cells with western blotting. As a normalization control, β-actin band was used. **g** Interaction of LRP6 with GRP94^WT^, ^S306A^, and ^S306D^ mutants were checked after co-transfecting HEK293 cells. Immunoprecipitation was performed with anti-Myc antibody and immunoprecipitates were detected using indicated antibodies. As input, 10% of cell lysate was used for analysis.

GRP94 has been previously identified as a substrate for protein kinase CK2α in the ER. Its phosphorylation is closely associated with protein processing and glycosylation [34, 35]. Computational analysis of the amino acid sequence of GRP94 using four independent predictive algorithms indicated that CK2α had the highest likelihood of phosphorylating serine (S) 306 and threonine (T) 786 residues of GRP94 (Supplementary Table S4). To investigate the interaction between GRP94 and CK2α, we designed a co-IP assay with transient transfection. Results shown in Fig. 3a demonstrated a clear interaction between GRP94 and CK2α. Furthermore, co-expression of GRP94 and CK2α in HEK293 cells resulted in a dose-dependent increase in GRP94 phosphorylation, as evidenced by western blot analysis (Fig. 3b). Conversely, knockout of CK2α failed to detect GRP94 phosphorylation at Ser residue (Fig. 3c). To determine the importance of CK2α kinase activity, we generated a kinase-dead CK2α mutant (K68M). As expected, the K68M mutant of CK2α failed to induce GRP94 phosphorylation (Fig. 3d). Interestingly, a serine-to-alanine (S306A) phosphorylation-null mutation in GRP94 failed to increase GRP94 phosphorylation levels by CK2α (Fig. 3e), indicating that CK2α phosphorylated GRP94 at the specific S306 residue. We then found that GRP94 was decreased in CK2α-depleted MDA-MB231 cells, suggesting that phosphorylation of GRP94 at S306 by CK2α could enhances GRP94 protein stability (Fig. 3f and Supplementary Fig. S3a).

Next, we investigated whether CK2α-mediated phosphorylation of GRP94 at S306 influenced the co-localization of GRP94 and LRP6 proteins. We generated a CK2α^KO^ MDA-MB231 cell line using Crispr-Cas9 system and re-expressed a phosphor-null mutant GRP94 (S306A) or a phosphor-mimetic mutant (S306D). Immunofluorescence analysis indicated that the co-localization of GRP94 and LRP6 was reduced in response to CK2α depletion or ectopic expression of GRP94-S306A in MDA-MB231 cells. However, overexpression of GRP94-S306D rescued the decrease co-localization and expression of LRP6 (Supplementary Fig. S3b). Consistent with a previous study showing that CK2α phosphorylation of Cortactin could affect its binding affinity for the Arp2/3 complex [36], we investigated whether CK2α phosphorylation of GRP94 might similarly impact its binding affinity for LRP6. To address this, we performed a co-IP assay by transfecting HEK293T cells with LRP6 and GRP94-WT, S306A, or S306D. As shown in Fig. 3g, GRP94-WT or S306D increased the binding affinity for LRP6, whereas S306A mutant showed no interaction with LRP6. However, GRP94-ΔT786 mutant and WT showed no difference in binding affinity with LRP6 (Supplementary Fig. S3c). These results collectively demonstrate that CK2α–mediated phosphorylation of GRP94 at S306 is crucial for GRP94 protein stabilization and optimal interaction with LRP6.

### CK2α-mediated GRP94 phosphorylation is essential for TNBC metastasis

**Fig. 4:**
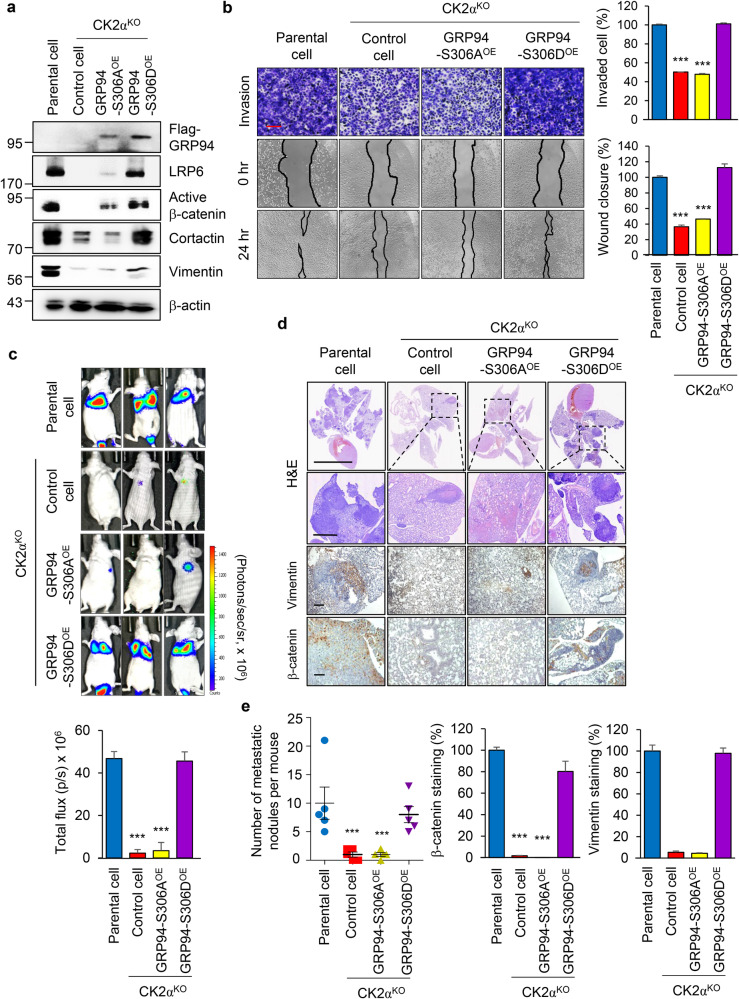
CK2α-mediated GRP94 phosphorylation promotes TNBC metastasis. **a** Western blotting analysis of Wnt signaling-related markers in CK2α^KO^ MDA-MB231 cells with stably expressing GRP94-S306A or S306D mutants was performed. As a normalization control, β-actin band was used. **b** Transwell and wound healing assays were performed using CK2α^KO^ MDA-MB231 cells stably expressing GRP94-S306A or S306D mutants. The histogram shows average percentage of migrated and invaded cells per view (right panel). ***p < 0.001 (Student’s *t* test). Scale bar = 200 μm. **c** Representative bioluminescence images are presented after tail vein injection of CK2α^KO^ MDA-MB231 cells with stably expressing GRP94-S306A or S306D mutants (1 × 10^6^) into nude mice as indicated (5 mice/group). Luciferase activity was detected using IVIS spectrum after luciferin injection. Luminescence intensities of total flux of photons in metastatic sites in each group are presented as mean ± SD (bottom panel). ***p < 0.001 (Student’s *t* test). Hematoxylin and eosin (H&E) (Scale bar = 200 μm (upper panel), 20 μm (bottm panel) and immunohistochemical staining of human Vimentin and β-catenin (Smale bar = 200 μm) in metastatic lung tumors (**d**) and quantification results of metastatic nodules and β-catenin expression (**e**). Data are expressed as mean ± SD. ***p < 0.001 (Student’s *t* test).

To elucidate whether CK2α-mediated phosphorylation of GRP94 at S306 was responsible for CK2α-induced invasion and migration of breast cancer cells, we assessed expression levels of key factors related to Wnt signaling and EMT in CK2α-depleted or GRP94 mutants expressing MDA-MB231 cells. Results revealed that expression levels of LRP6, active β-catenin, Cortactin, and Vimentin were downregulated in MDA-MB231 cells with CK2α knocked out and GRP94-S306A-overexpressing MDA-MB231 cells, while expression levels of these markers were restored in GRP94-S306D-overexpressing cells (Fig. 4a). However, deletion of T786 residue in GRP94-S306D construct did not significantly impact the level of LRP6 (Supplementary Fig. S4a). These results suggest that GRP94-induced LRP6 stabilization is mainly associated with CK2α-mediated phosphorylation of S306, not T786.

Consistent with above results, overexpression of CK2α in MCF7 cells increased Wnt signaling and EMT-related markers, whereas overexpression of a kinase-dead CK2α mutant (K68M) did not significantly affect these markers. Importantly, overexpression of GRP94-S306D in CK2α-K68M-overexpressing MCF7 cells resulted in significant increases of Wnt signaling and EMT-related markers (Supplementary Fig. S4b). Moreover, deletion of T786 residue in GRP94 did not alter the level of LRP6 in MCF7 cells (Supplementary Fig. S4c). Correspondingly, transwell invasion and wound scratch assays demonstrated weakened invasion and migration abilities of CK2α^KO^ and GRP94-S306A^OE^ MDA-MB231 cells, whereas overexpression of GRP94-S306D restored metastatic abilities of CK2α^KO^ cells to levels similar to those of parental cells (Fig. 4b). Similarly, overexpression of CK2α in MCF7 cells enhanced invasion and migration, whereas overexpression of the CK2α-K68M mutant did not affect these abilities as expected. However, restoration of GRP94-S306D rescued metastatic abilities of CK2α-K68M^OE^ MCF7 cells, resembling the metastatic ability of CK2α-WT^OE^ MCF7 cells (Supplementary Fig. S4d).

Importantly, to investigate the impact of GRP94-S306 phosphorylation on breast cancer metastasis in vivo, we performed intravenous injections of luciferase-expressing MDA-MB231 parental cells, CK2α^KO^, GRP94-S306A or S306D-restored cells into nude mice. Consistent with in vitro findings, bioluminescence imaging revealed that lung metastasis of MDA-MB231 cells was significantly inhibited in CK2α^KO^ and GRP94-S306A mutant-reexpressing cells. Such inhibition was rescued by re-expression of GRP94-S306D (Fig. 4c, d). The number of metastatic nodules and β-catenin expression levels were also markedly reduced in CK2α depletion and GRP94-S306A mutant expressing lung tissues (Fig. 4e). Similarly, overexpression of CK2α-WT significantly induced lung metastasis of MCF7 cells, whereas overexpression of the CK2α-K68M mutant had no effect. However, restoration of GRP94-S306D rescued the metastatic ability of CK2α-K68M^OE^ MCF7 cells (Supplementary Fig. S5a, b). Consistent with these results, the number of metastatic nodules and intensity of β-catenin in lung tissues were markedly increased in CK2α-WT^OE^ or GRP94-S306D^OE^ MCF7-injected lung tissues (Supplementary Fig. S5c). These results provide strong evidence that CK2α can promote metastasis of breast cancer cell through S306 residue specific phosphorylation of GRP94.

### Benzamidine, a novel CK2α inhibitor, reduces LRP6 stability by suppressing GRP94 phosphorylation

**Fig. 5:**
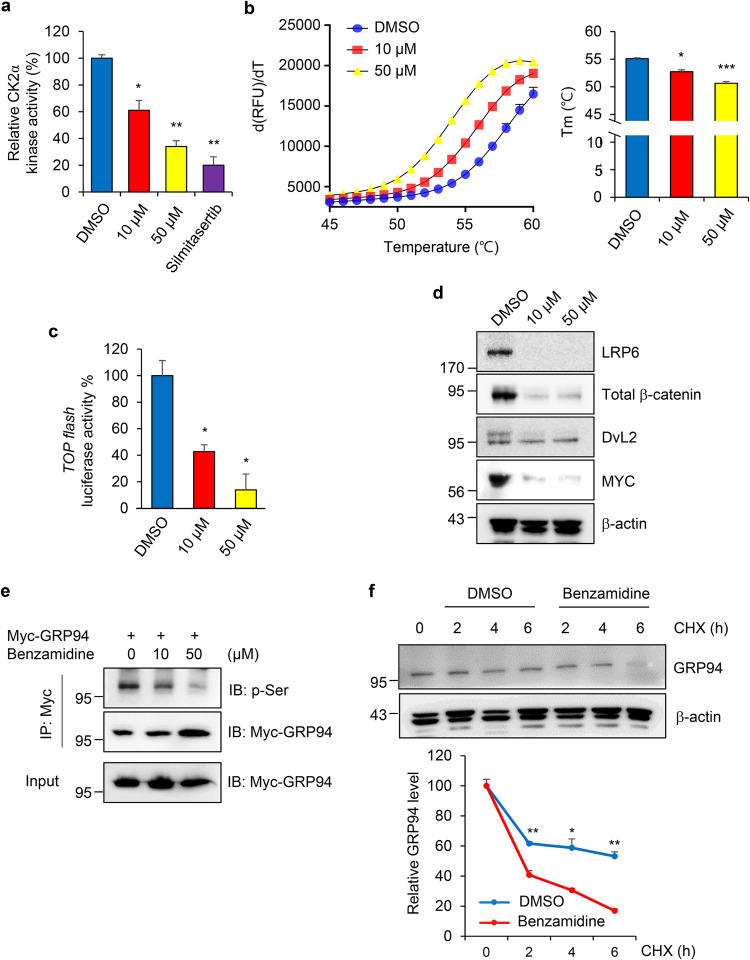
Benzamidine, a novel CK2α inhibitor, reduces LRP6 stability by suppressing GRP94 phosphorylation. **a** CK2α kinase activity was measured after treatment with vehicle, benzamidine (10 μM and 50 μM), or silmitasertib (20 μM). Cell lysates were used for in vitro kinase assay to detect the intensity of phosphorylated substrate. Data are presented as relative activity of vehicle-treated sample. *p < 0.05; **p < 0.01 (Student’s *t* test). **b** Thermal shift assay for CK2α was performed after addition of benzamidine (10 and 50 μM). Positive derivative (d(RFU)/dT) curves are shown for control and treated with benzamidine (10 and 50 μM). Midpoint temperatures of protein-unfolding transition (*T*_m_) are presented as bars. Values are presented as mean ± SD of at least three independent measurements. *p < 0.05; ***p < 0.001. One-way ANOVA was followed by Dunnett’s multiple comparisons test. **c** MDA-MB231 cells were seeded into a 12-well plate and transfected with TOPflash/FOPflash reporter plasmids. After 24 h, cells were treated with benzamidine (10 and 50 μM) or vehicle for an additional 24 h. β-catenin transcriptional activity was assessed by measuring luminescence. TOPflash values are presented as fold change from control cells. β-galactosidase activity was used as a normalization control. Bars represent mean ± SD. *p < 0.05 (Student’s *t* test). **d** Wnt signaling-related markers were checked in MDA-MB231 cells treated with benzamidine (10 and 50 μM) for 24 h. β-actin band was used as a normalization control. **e** MDA-MB231 cells were transfected with Myc-GRP94 and treated with increasing concentrations of benzamidine. Cell extracts were then subjected to immunoprecipitation with anti-Myc antibody and detected with anti-p-Ser antibody. **f** MDA-MB231 cells were treated with benzamidine (50 μM). Stability of GRP94 and LRP6 was measured after adding CHX for the indicated time course, followed by western blotting analysis. Results are representative of at least three independent experiments and data are presented as mean ± SD.

In our previous study, we developed a screening strategy for identification of novel metastasis inhibitor based on invadopodia staining [37]. Among candidate compounds, benzamidine as a potential CK2α inhibitor [38, 39] exhibited the highest inhibitory activity against invadopodia formation. To confirm the inhibitory activity of benzamidine on CK2α, we checked kinase activity using an in vitro assay system. As shown in Fig. 5a, benzamidine had a strong inhibitory effect on CK2α in a dose-dependent manner compared with silmitasertib. Next, we determined the inhibitory effect of benzamidine on melting temperature (*T*_m_) of CK2α protein using a TSA system, a widely used method for determining target engagement by detecting changes in a protein’s thermal stability upon drug binding. Normally, CK2α has a *T*_m_50 of 55.10° (95% CI: 53.35–56.49). However, addition of benzamidine decreased the thermal stability *T*_m_50 of CK2α to 52.74° (95% CI: 50.66–54.32) or 50.64° (95% CI: 48.28–52.38) depending on the increasing amount (Fig. 5b). These results suggest that benzamidine potentially makes CK2α less stable, causing it to denature at a lower temperature.

Moreover, treatment with benzamidine significantly diminished TOPflash luciferase activity, indicating attenuation of Wnt signaling in MDA-MB231 cells (Fig. 5c). To confirm the involvement of benzamidine in Wnt signaling pathway, MDA-MB231 cells were treated with benzamidine and Wnt signaling-associated proteins. Expression levels of Wnt-related proteins were markedly reduced in response to benzamidine treatment (Fig. 5d). Subsequently, we investigated whether inhibition of CK2α kinase activity by benzamidine might affect GRP94 phosphorylation. Western blot analysis revealed that benzamidine strongly decreased phosphorylation of GRP94 at Ser residue (Fig. 5e). Furthermore, we conducted a CHX assay to examine the effect of benzamidine on LRP6 and GRP94 protein stability. Results demonstrated that treatment with benzamidine decreased the stability of both LRP6 and GRP94 proteins in MDA-MB231 cells compared to the control (no treatment) (Fig. 5f). Collectively, our findings indicate that inhibition of CK2α activity by benzamidine can suppress GRP94 phosphorylation and lead to destabilization of LRP6 protein, resulting in inhibition of the Wnt signaling pathway.

### Benzamidine suppresses EMT process and metastasis of TNBC

**Fig. 6:**
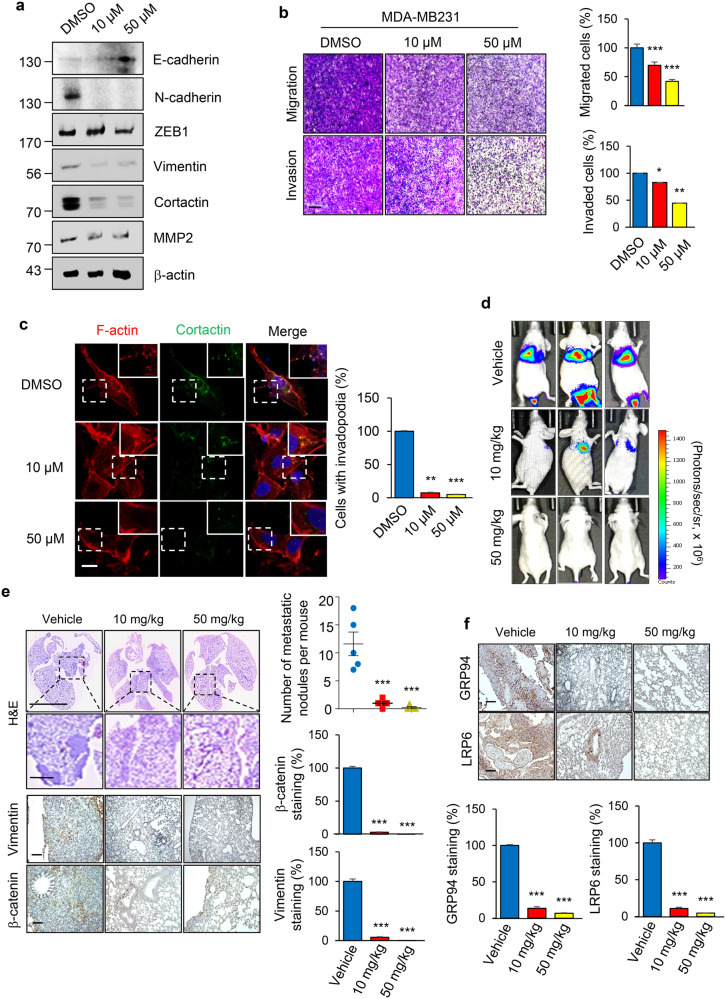
Benzamidine suppresses EMT and metastasis of TNBC. **a** EMT-related markers (E-cadherin, N-cadherin, ZEB1, and Vimentin), metastasis-related protein (MMP2), and invadopodia-related protein (Cortactin) were checked in MDA-MB231 cells after treatment with benzamidine (10 and 50 μM) or vehicle for 24 h. β-actin band was used as a normalization control. **b** Transwell migration and invasion assays were performed in MDA-MB231 cells after treatment with benzamidine (10 and 50 μM) for 24 h. The histogram shows relative percentage of migrated and invaded cells compared to control cell (right panel). Scale bar = 200 μm. *p < 0.05; **p < 0.01; ***p < 0.001 (Student’s *t* test). **c** Representative immunofluorescence images showing invadopodia in MDA-MB231 cells treated with benzamidine (10 and 50 μM) for 24 h were revealed by F-actin and Cortactin double staining. The inset shows a magnified image of the invadopodia in the box. The percentage of cells with invadopodia in MDA-MB231 cells was quantified and compared with control cells. Scale bar = 50 μm. **p < 0.01; ***p < 0.001 (Student’s *t* test). **d** Representative bioluminescence images were presented after tail vein injection of MDA-MB231-luc cells (1 × 10^6^) into nude mice. Mice bearing tumor cells were divided into three comparable groups (5 mice/group). After another 1 week, benzamidine (10 and 50 mg/kg) was administered intraperitoneally every 3 days. Luciferase activity was detected after 6 weeks using the IVIS spectrum after luciferin injection. **e** Hematoxylin and eosin (H&E) (Scale bar = 200 μm (upper panel), 20 μm (bottm panel)) and immunohistochemical staining of human Vimentin and β-catenin (Scale bar = 200 μm) were performed in MDA-MB231 cells treated with benzamidine (10 and 50 mg/kg) or vehicle in metastatic lung tumors. Quantification of metastatic nodules and β-catenin, Vimentin expression is shown. Scale bar = 200 μm. Data are expressed as mean ± SD. ***p < 0.001 (Student’s *t* test). **f** Immunohistochemical staining of GRP94 and LRP6 was performed with metastatic lung tissues after injection with benzamidine (10 and 50 mg/kg). Quantification of GRP94 and LRP6 expression was done with ImageJ software. Scale bar = 200 μm. Data are expressed as mean ± SD. ***p < 0.001 (Student’s *t* test).

To investigate the inhibitory effect of benzamidine on EMT and metastasis of TNBC, we first examined expression levels of EMT- and metastasis-related markers in benzamidine-treated MDA-MB231 cells. Western blotting and qRT-PCR results revealed that benzamidine increased the expression of E-cadherin dose-dependently but decreased protein and mRNA levels of N-cadherin, ZEB1, Vimentin, Cortactin, and MMP2 (Fig. 6a and Supplementary Fig. S6a). Interestingly, benzamidine did not affect the proliferative capacity of breast cancer cells (Supplementary Fig. S6b). To further assess the impact of benzamidine on the metastatic capacity of breast cancer cells, we performed transwell migration and invasion assays, which revealed that benzamidine suppressed migration and invasion abilities of TNBC cells (Fig. 6b). Additionally, benzamidine markedly reduced the invadopodia formation and gelatin degradation of TNBC cells (Fig. 6c and Supplementary Fig. S6c).

Furthermore, we evaluated effect of benzamidine on metastasis of breast cancer cell using a tail vein injection model. At four weeks after benzamidine treatment, metastatic dissemination of MDA-MB231 cells was monitored by bioluminescence imaging. We observed strong fluorescent signals in lungs of the vehicle group, whereas a much weaker signal was detected in the group with benzamidine treatment (Fig. 6d and Supplementary Fig. S6d). Additionally, histological analysis with H&E and IHC staining revealed decreased staining for Vimentin, β-catenin, GRP94, and LRP6 in benzamidine-treated groups compared to those in the vehicle-treated group (Fig. 6e, f). Collectively, these findings indicate that benzamidine can act as a novel inhibitor of EMT and metastasis of TNBC by blocking CK2α-mediated GRP94 phosphorylation.

## Discussion

**Fig. 7:**
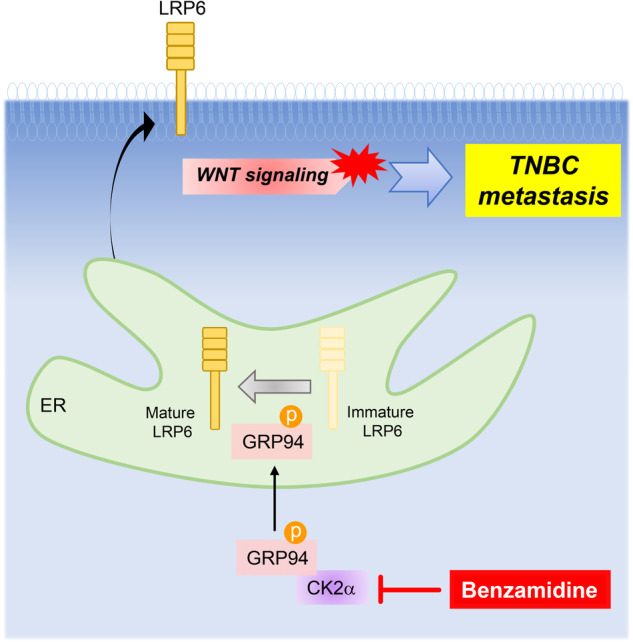
Schematic diagram illustrating the important role of CK2α-GRP94-LRP6 axis in TNBC metastasis. In TNBC cells, elevated CK2α phosphorylates GRP94 at serine 306 residue, leading to increased stability of the GRP94 protein. As an ER chaperone, GRP94 promotes the maturation of LRP6 protein, resulting in activation of Wnt signaling. Collectively, the activation and upregulation of CK2α and GRP94 synergistically promote the metastasis of TNBC cells through the Wnt signaling pathway. By targeting CK2α-mediated phosphorylation of GRP94 with benzamidine, metastasis of TNBC can be effectively controlled as a therapeutic strategy.

Mechanistic insights obtained in this study elucidated molecular mechanisms through which GRP94 promoted TNBC metastasis (Fig. 7). Specifically, CK2α, an active kinase in advanced breast cancer, phosphorylated GRP94 at a conserved serine 306 (S306) residue. This phosphorylation event enhanced the stability of GRP94 and strengthened its interaction with LRP6, a key component of the canonical Wnt signaling pathway. Consequently, the activation of canonical Wnt signaling contributed to the metastatic potential of TNBC cells. Importantly, the identification of benzamidine, a novel CK2α inhibitor that can effectively suppress GRP94 phosphorylation and LRP6 stabilization, provides a promising avenue for therapeutic interventions targeting this critical pathway.

Based on results of this study, targeting GRP94 and CK2α holds significant promise for the development of novel therapeutic strategies against TNBC metastasis. Besides as a chaperone’s activity, GRP94 is closely associated with tumorigenesis and metastasis of various cancers. Increased GRP94 level generally correlates with a higher T-stage (*p* < 0.05) and a shorter overall survival of multiple myeloma patients [40]. GRP94 expression is also associated with increased tumorigenesis and metastasis of breast cancer. Especially, abnormal overexpression of GRP94 has been implicated in brain metastasis by promoting pro-survival autophagy [41]. Moreover, downregulation of GRP94 in prostate cancer cells can cause upregulation of cleaved caspase-9 and Bax expression levels, while suppression of Bcl-2 and Vimentin expression levels can induce apoptosis and inhibition of prostate cancer cell invasion [42]. Thus far, several studies have been performed to identify specific GRP94 inhibitors as cancer therapeutics [43–45]. Although no drugs are successful clinical trials, it is a valuable approach to develop inhibitors that can specifically suppress GRP94 expression or function to control invasive properties and metastatic capacity of TNBC cells.

In our study, the use of benzamidine, a CK2α inhibitor, presents a targeted approach to disrupt phosphorylation-mediated activation of GRP94 and the downstream canonical Wnt signaling pathway, leading to suppression of metastatic activity of TNBC (Figs. 5, 6). These therapeutic interventions have potential to improve patient outcomes by reducing metastasis and preventing disease progression. CK2α is a highly conserved serine/threonine protein kinase that plays diverse cellular functions in the majority of cancer progression, including the regulation of cell proliferation, survival, and metastasis [46]. Furthermore, CK2α can be used as an important diagnostic and prognostic marker in some malignancies, suggesting that CK2α is a potential anticancer target. CK2α inhibitors have been investigated. As representative agents, CX-4945 and CIGB-300 are currently undergoing phase I/II clinical trials in malignancies [47–50]. Through identification of novel inhibitor or development of pre-identified drug derivatives, CK2α inhibitor can be applied as promising therapeutics against metastasis of TNBC [51, 52].

In conclusion, our findings highlight the therapeutic potential of GRP94 and/or CK2α inhibitors for suppressing EMT and metastasis of TNBC. The development and optimization of GRP94 and/or CK2α inhibitors hold promise for improving clinical outcomes of patients with TNBC by targeting key drivers of cancer progression and metastasis. Further investigations and clinical trials are warranted to fully evaluate the efficacy and safety of GRP94 and/or CK2α inhibitors as novel treatment modalities for TNBC.

## Materials and methods

### Cell culture and reagents

TNBC cell lines (MDA-MB-231, Hs578T, and MDA-MB-157) were maintained using Dulbecco’s Modified Eagle’s Medium including penicillin/streptomycin, and 10% FBS (ATCC, Manassas, VA, USA). Non-TNBC cell lines (BT474, MCF7, and T47D) were cultured in RPMI-1640 supplemented with antibiotics and 10% FBS at 37 °C in a 5% CO_2_ incubator. Cell lines were periodically checked for mycoplasma contamination with PCR-based protocol. Antibodies used for immunoblotting and immunoprecipitation are listed in Supplementary Table S1. Recombinant CK2α protein was obtained from Origene (TP760181, Rockville, MD, USA). Benzamidine (12072) and cycloheximide (CHX, C7698) were purchased from Sigma-Aldrich (St. Louis, MO, USA). Silmitasertib (CX-4945, HY-50855) was obtained from MedChemExpress Biotech Co., Ltd. (Monmouth Junction, NJ, USA).

### RNA preparation and real-time PCR

Total RNA was isolated using Trizol (Invitrogen, Carlsbad, CA, USA) reagent according to the manufacturer’s instructions. Then, RNA was transformed to cDNA with reverse transcription using a PrimeScript II RT Reagent Kit (Takara, Kyoto, Japan). Quantification of specific gene expression was measured with SYBR premix Ex Taq™ (Takara) and a Prism 7900HT sequence detection system (Thermo Fisher Scientific, Rockford, IL, USA) following the manufacturer’s protocols. Relative gene expression was determined using the 2^ΔCt^ (internal control) − ΔCt (gene) method and normalized to cyclophilin [53]. Data was acquired from at least triplicate experiments and denoted as mean ± standard deviation (SD). Used primer sequences are listed in Supplementary Table S2.

### Immunoblotting and immunoprecipitation

Cells were harvested with lysis buffer (50 mM Tris (pH 7.5), 150 mM NaCl, 10% glycerol, 0.5% Nonidet P-40, and protease inhibitors) for 30 min at 4 °C. After removal of cell debris, protein concentrations were measured with the bicinchoninic acid method (Thermo Fisher Scientific). Equal amounts of protein samples were then separated by sodium dodecyl sulfate (SDS)–polyacrylamide gel electrophoresis, followed transfer to Immobilon PVDF membrane (Millipore, Bedford, MA, USA), and incubated with appropriate primary antibodies. Target proteins were detected using chemiluminescent reagents (Thermo Fisher Scientific) according to the manufacturer’s protocols [54]. For immunoprecipitation, protein samples were mixed with appropriate antibodies for overnight at 4 °C and collected with addition of protein A/G-agarose beads (Santa Cruz, Dallas, TX, USA) after incubation at 4 °C for another 1 h. Interacted protein complexes were harvested with 2x SDS sample buffer and analyzed with immunoblotting method.

### Wound healing and transwell invasion assay

To assess migration ability of breast cancer cells, cells were seeded into 12-well plates and allowed to reach confluency for ~24 h [55]. After scratch was created using a pipette tip, migration ability was analyzed under a microscope observation. To evaluate the invasion ability, same number of cells (5 × 10^4^) were seeded into the upper chamber (8 μm inserts; Thermo Scientific) with serum-free media while the lower chamber was filled with 10% FBS medium. After 24–36 h, cells in the upper chamber were removed and fixed with 4% paraformaldehyde (PFA) and visualized with 0.05% crystal violet (Sigma Aldrich). Representative images were captured using a confocal microscopy (LSM710, Carl Zeiss, Oberkochen, Germany). Total number of invaded cells was measured with ImageJ program (National Institutes of Health, Bethesda, MD, USA).

### Immunofluorescence and immunohistochemistry staining protocol

For immunofluorescence staining, cells were seeded into 4-well chamber slides. After 24 h of incubation, cells were fixed with 4% PFA for 15 min at room temperature. Then, stained cells were washed with PBS three times and permeabilized with 1% Triton X-100/PBS. After additional washing steps, cells were treated with blocking buffer (1% BSA in PBS) for 1 h. Primary antibodies (1:100) were then applied followed by incubation at 4 °C overnight. Following removal of the primary antibodies and subsequently cells were treated with Alexa Fluor 594-conjugated (A32740, Invitrogen) and Alexa Fluor 488-conjugated (A32723, Invitrogen) secondary antibodies for 1 h at room temperature. Then, nuclei were counterstained with 4′,6-diamidino-2-phenylindole (DAPI) for 1 min. Fluorescence images were analyzed with a confocal microscope and quantified using ImageJ software [56].

For immunohistochemistry (IHC) staining, lung tissues were fixed in 4% neutral-buffered formalin, embedded, and sectioned as described previously [57]. Slides were treated with 0.03% hydrogen peroxide, microwaved in a 10 mM citrate buffer (pH 6.0) containing 0.01% Tween 20 for 10 min, and then incubated with appropriate antibodies. Antibodies were analyzed with diaminobenzidine (DAB) reagent, while the nuclei were counterstained with hematoxylin QS (H-3404; Vector Laboratories, Burlingame, CA, USA). Random images of stained slides were acquired with Motic Easyscan Digital Slide Scanner (Motic Hong Kong Limited, Hong Kong, China).

### PCR-based site-directed mutagenesis

To generate CK2α-K68M (kinase dead) and GRP94-S306A (dephosphorylation mutant of GRP94, serine at position 306 mutated to alanine) or S306D (phosphor-mimetic mutation of GRP94, serine at position 306 mutated to aspartic acid), PCR-based site-directed mutagenesis method was used as described previously [58]. Point mutations were inserted into each DNA construct with overlapping extension PCR protocol and the mutated PCR product was cloned into the plasmid pCAG-Flag vector. Then, mutation results were confirmed by sequencing. To remove potential phosphorylation site of T786 residue in GRP94, the plasmid expressing the T786 deletion mutant was generated by PCR-mediated amplification of pCS4-Flag-GRP94 wild type construct with appropriate primer. PCR products were digested with restriction enzymes and cloned into pCAG vector. Primer sets used for mutagenesis are listed in Supplementary Table S3.

### TCF luciferase reporter assay for Wnt/β-catenin signaling

To evaluate the activating role of GRP94 on Wnt signaling, GRP94-knockouted or parental MDA-MB231 cells were transfected with the TCF reporter plasmid (TOPFlash) and β-galactosidase plasmid as a control to monitor the transfection efficiency. Transfection was performed with lipofectamine 2000 (Invitrogen) according to the manufacturer’s protocol. After 48 h, the cells were lysed and assayed for luciferase and β-galactosidase activity using Dual-Luciferase Reporter Assay System (Promega, Madison, WI, USA), according to the manufacturer’s instructions [59]. All assays were performed in triplicate using a Victor^2^ luminometer (Perkin Elmer, Boston, MA).

### Generation of knockout and overexpression of breast cancer cell lines

To generate GRP94 and CK2α knockout (KO) cell lines, the following sgRNA sequences were cloned into the LentiCRISPRv2 vector (AddGene, Watertown, MA, USA): 5′-CGGATGATGAAGTAGTACAG-3′ for GRP94 sgRNA and 5′-GAACTGACCTGACATCATAT-3′ for CK2α sgRNA [60]. For overexpression (OE), Flag-CK2α-WT, CK2α-K68M, GRP94-WT, GRP94-S306A, GRP94-S306D, GRP94-ΔT786, and GRP94-S306D/ΔT786 DNAs were cloned into the pCAG lentiviral vector. Lentivirus production was performed by transfecting HEK293T cells with lentiviral packaging plasmids, psPAX2 and pMD2.G (AddGene). After 48 h, viral supernatants were harvested, filtered through a 0.45 μm syringe filter, and stored at −80 °C for future use.

### Fluorescent gelatin degradation assay

Activity of invadopodia was measured using a QCMTM gelatin degradation assay kit (ECM670, Millipore, Burlington, MA, USA) according to manufacturer’s protocols [61]. Glass-bottom 8-well dishes were incubated with 0.1 mg/ml poly l-lysine and crosslinked with 0.5% glutaraldehyde. Then, dishes were coated with Oregon Green 488-conjugated gelatin at 37 °C for 1 h. After treatment with sodium borohydride and ethanol, medium was added to dishes. After plating cells followed by 48 h of incubation, cells were immobilized with 4% PFA, permeabilized with 0.5% Triton X-100 following blocking with 3% bovine serum albumin in PBS and incubated with primary antibody against F-actin/Phalloidin (Invitrogen) overnight at 4 °C. Then, nuclei were counterstained with DAPI for 1 min before observation.

### CK2 in vitro kinase assay

CK2 activity in breast cancer cells was analyzed using a Cyclex CK2 Kinase Assay/Inhibitor Screening kit (MBL International Corp., Woburn, MA, USA) following the manufacturer’s protocols. MDA-MB231 cells were treated with benzamidine (10 μM or 50 μM) or silmitasertib (20 μM) for 24 h. After lysis with kinase reaction buffer, samples were moved into 96-well plates pre-coated with a substrate of CK2α (recombinant p53 amino acids 1–55) and incubated at 30 °C for 10 min in the presence of ATP. These wells were washed and treated with HRP-conjugated antibody and substrate reagent. The reaction was terminated by adding TK-4D4 and TMB. The color was quantified using a 96-well plate reader at 450 nm/540 nm. Data were acquired at least triplicate experiments and statistical significance was set at *p* < 0.05.

### Thermal shift assay

Thermal shift binding assay (TSA) was performed with a Bio-Rad CFX real-time PCR system (Hercules, CA, USA). CK2α protein was equilibrated in a buffer (pH 7.5, 100 mM Tris-HCl, 100 mM NaCl) with SYPRO Orange dye (Invitrogen) after addition of DMSO or benzamidine. Samples were freshly prepared and dispensed into 384-well PCR plates at a final volume of 10 μL per well. Fluorescence intensity of each well was measured with a temperature gradient range from 25 to 95 °C with a heating rate increase of 1 °C per minute. To determine the effect of benzamidine on melting temperature (*T*_m_) of CK2α protein, a Boltzmann model was used to generate the protein unfolding curves with GraphPad Prism (v10.0) software.

### In vivo metastasis animal model

Xenograft tumor models were constructed using 6-8-week-old immunodeficient BALB/c athymic female nude mice (Orient Bio, Seongnam, Korea). Animal experiments followed the guidelines approved by Institutional Animal Care and Use Committee-approved protocols of the Lee Gil Ya Cancer and Diabetes Institute, Gachon University (#LCDI-2019-0146, Incheon, Korea). MDA-MB231 cells stably expressing luciferase and CK2α^KO^, CK2α^KO^/GRP94-S306A^OE^, and CK2α^KO^/GRP94-S306D^OE^ or MCF7 cells stably expressing luciferase with CK2α^OE^, CK2α-K68M^OE^, and CK2α-K68M^OE^/GRP94-S306D^OE^ (1 × 10^6^ cells in 100 μl) were intravenously injected into nude mice (five animals per group) [62].

To evaluate the effect of benzamidine, a CK2α inhibitor, on metastasis of TNBC cells, MDA-MB231 cells stably expressing luciferase were intravenously injected into nude mice (five animals per group). Mice were blindly randomized into three groups and treated with benzamidine (10 mg/kg or 50 mg/kg) or DMSO by intraperitoneal injection every other day. Metastasis intensity into lung was monitored with in vivo optical imaging system (Caliper Life Sciences, Hopkinton, MA, USA) at the Core Facility for Cell In-vivo Imaging of Gachon University weekly by intraperitoneal injection of luciferin reagent (150 mg/ml). After 6 weeks, mice were sacrificed and lung tissues were fixed with 10% formalin, processed, and embedded in paraffin. Prepared lung slides were analyzed with hematoxylin and eosin (H&E) and IHC staining with Vimentin and β-catenin antibodies. Metastatic areas were calculated with ImageJ software.

### Statistics

All statistical results are presented as mean ± standard deviations (SDs). Student’s *t* test or one-way ANOVA was performed using GraphPad Prism 10.0 software. The sample size for each statistical analysis was indicated. Differences with *p* < 0.05 were considered statistically significant (NS, not significant; *p < 0.05; **p < 0.01; ***p < 0.001).

## Supplementary information

Supplementary Figures and Tables

Original Data File

## Data Availability

All data generated in this study is available in the main text or the supplementary materials. Any additional information required to reanalyze the data reported in this paper is available from the lead contact upon request.
