# Correction: CK2α-mediated phosphorylation of GRP94 facilitates the metastatic cascade in triple-negative breast cancer

**DOI:** 10.1038/s41420-025-02298-y

**Published:** 2025-02-10

**Authors:** Hye-Youn Kim, Young-Mi Kim, Suntaek Hong

**Affiliations:** Department of Biochemistry, Lee Gil Ya Cancer and Diabetes Institute, Gachon University College of Medicine, Incheon, 21999 Republic of Korea

**Keywords:** Breast cancer, Metastasis, Predictive markers

Correction to: *Cell Death Discovery* 10.1038/s41420-024-01956-x, published online 22 April 2024

In this article the funding note “This work was supported by the National Research Foundation of Korea (NRF) grant funded by the Korea government (MSIT) (RS-2023-00246400) and the Basic Science Research Capacity Enhancement Project through Korea Basic Science Institute (2021R1A6C101A432) to SH, the Basic Science Research Program funded by the Ministry of Education (2022R1I1A1A01069333) to YMK and the NRF grant funded by the Korea government (MSIT) to HYK (2019R1C1C1006055).” has been updated to “This work was supported by the National Research Foundation of Korea (NRF) grant funded by the Korea government (MSIT) (RS-2023-00246400) and the Basic Science Research Capacity Enhancement Project through Korea Basic Science Institute (2021 R1A6C101A432) to SH, the Basic Science Research Program funded by the Ministry of Education (2022R1I1A1A01069333) to YMK and the NRF grant funded by the Korea government (MSIT) to HYK (RS-2022-NR072269).”

The original article has been corrected.

