# Supplementary material for: CK2α-mediated phosphorylation of GRP94 facilitates the metastatic cascade in triple-negative breast cancer
Source: Cell Death Discov. 2024 Apr 22;10:185. doi: 10.1038/s41420-024-01956-x (PMC11035675; doi:10.1038/s41420-024-01956-x)
Supplement: Supplementary file 1 — Supplementary Figures and Tables [file 41420_2024_1956_MOESM1_ESM.pdf]

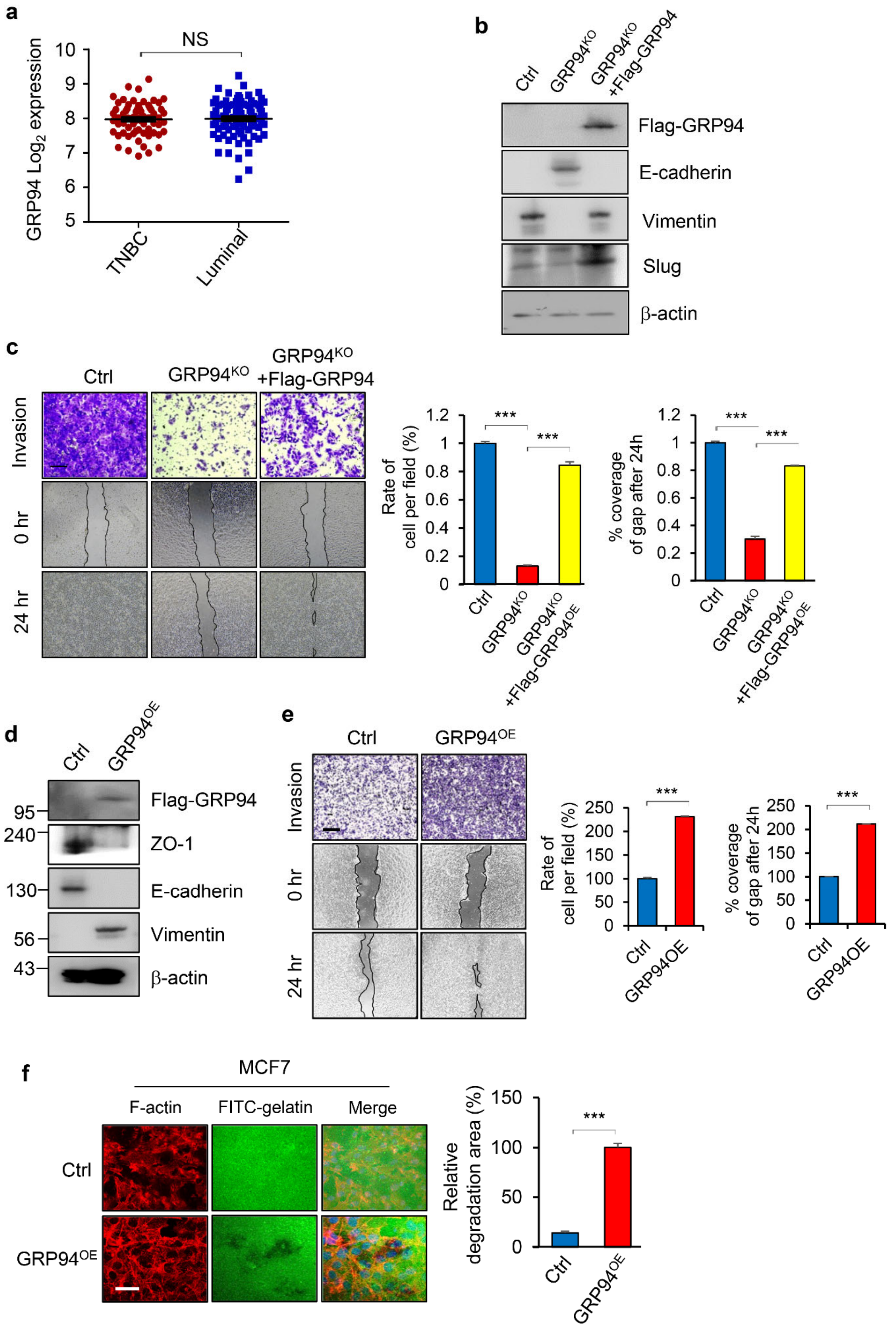

a
GRP94 Log2 expression
NS
10
9
8
7
6
5
TNBC
Luminal
b
Ctrl
GRP94KO
GRP94KO +Flag-GRP94
Flag-GRP94
E-cadherin
Vimentin
Slug
β-actin
c
Ctrl
GRP94KO
GRP94KO +Flag-GRP94
Invasion
0 hr
24 hr
Rate of cell per field (%)
% coverage of gap after 24h
1.2
1
0.8
0.6
0.4
0.2
0
***
Ctrl
GRP94KO
GRP94KO +Flag-GRP94OE
d
Ctrl
GRP94OE
95
240
130
56
43
Flag-GRP94
ZO-1
E-cadherin
Vimentin
β-actin
e
Ctrl
GRP94OE
Invasion
0 hr
24 hr
Rate of cell per field (%)
% coverage of gap after 24h
250
200
150
100
50
0
***
Ctrl
GRP94OE
f
MCF7
F-actin
FITC-gelatin
Merge
Ctrl
GRP94OE
Relative degradation area (%)
150
100
50
0
***
Ctrl
GRP94OE

Supplementary Fig. S1 GRP94 promotes EMT and metastasis of breast cancer cells. a Scatter dot plots displaying GRP94 mRNA levels in a panel of 52 breast cancer cell lines using public microarray datasets. NS, not significant. **b** Epithelial-mesenchymal transition markers (E-cadherin, Vimentin, and Slug) were validated in $GRP94^{KO}$ and Flag-GRP94-restored $GRP94^{KO}$ MDA-MB231 cells using western blotting. The β-actin band was used as a normalization control. **c** Transwell and wound healing assays were performed in in $GRP94^{KO}$ and Flag-GRP94-restored $GRP94^{KO}$ MDA-MB231 cells to assess the metastatic potential. The histogram represents the relative percentage of migrated and invaded cells compared to control cells. Scale bar = 200 μm. ***, $p < 0.001$ (Student's t-test). **d** Epithelial-mesenchymal transition markers (ZO-1, E-cadherin, and Vimentin) were validated in MCF7-$GRP94^{OE}$ cells and control cells using western blotting. β-actin band was used as a normalization control. **e** Transwell and wound healing assays were performed in MCF7-$GRP94^{OE}$ and control cells to assess the metastatic potential. The histogram represents the relative percentage of migrated and invaded cells compared to control cells. Scale bar = 200 μm. ***, $p < 0.001$ (Student's t-test). **f** Invasion activities of the indicated cells were evaluated by plating onto FITC-gelatin-coated slides. The intensity of FITC fluorescence was then measured. The degree of gelatin degradation was quantified using ImageJ software and compared to control cells (right panel). Scale bar = 50 μm. ***, $p < 0.001$ (Student's t-test). Data are presented as mean ± SD.

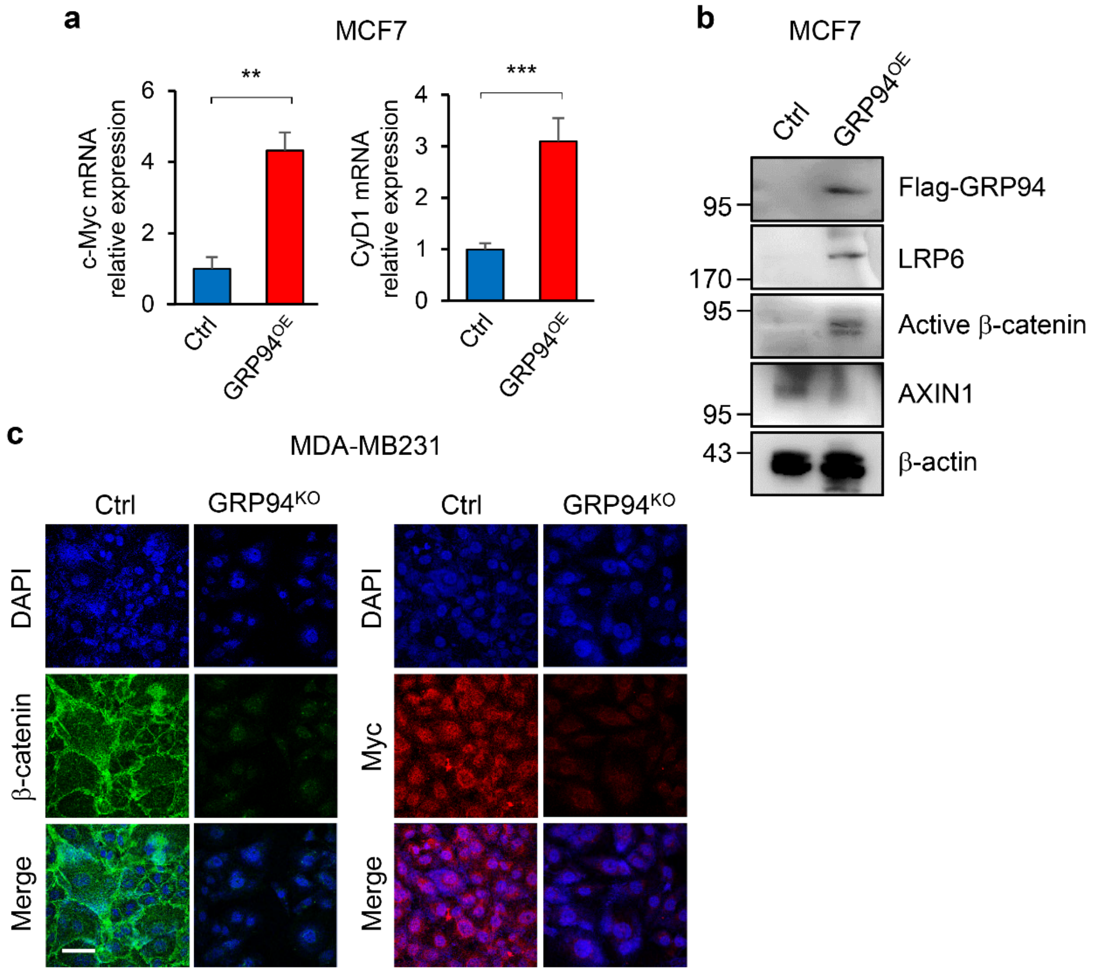

Supplementary Fig. S2 GRP94 activates Wnt signaling pathway. a mRNA expression levels of Wnt signaling-related markers (c-Myc and CyclinD1) were validated in MCF7-GRP94^{OE} and control cells using qRT-PCR. Cyclophilin was used as a normalization control. **, $p < 0.01$; ***, $p < 0.001$ (Student's t-test). **b** Wnt signaling-related markers were detected in MCF7-GRP94^{OE} and control cells with western blotting analysis. β-actin band was used as a normalization control. **c** Confocal immunofluorescence analysis for β-catenin and Myc was performed with MDA-MB231-GPR94^{KO} and control cells. Nuclei were detected with DAPI staining. Localization of proteins was analyzed by merging images. Scale bar = 50 μm. Data are presented as mean ± SD.

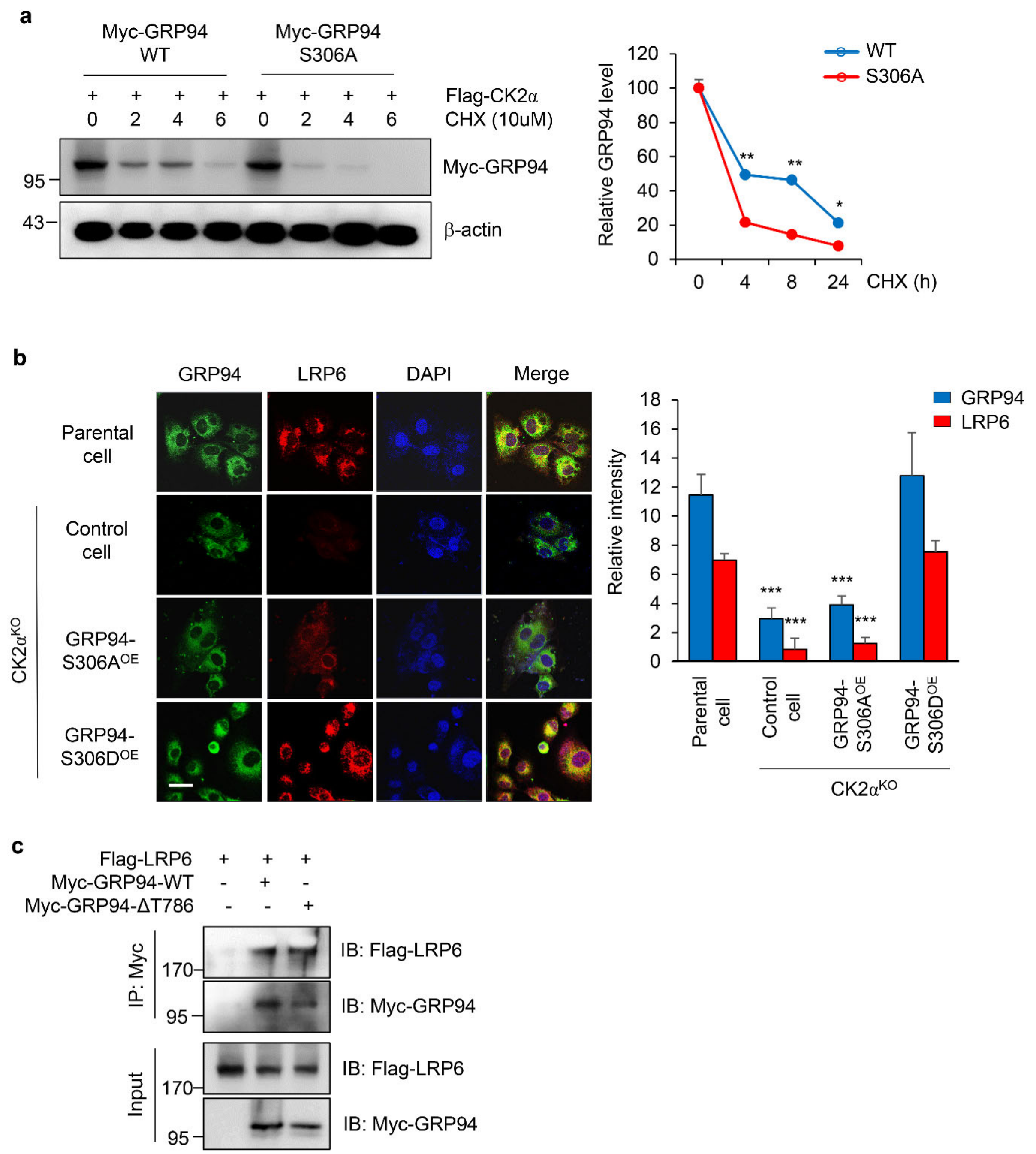

Supplementary Fig. S3 CK2α enhances GRP94 protein stability. a Stability of GRP94 was examined using western blotting in HEK293 cells co-transfected with Flag-CK2α or Myc-GRP94 WT or S306A mutant constructs, followed by incubation with 10 μM CHX for the indicated time (left panel). Relative GRP94 protein levels normalized to corresponding β-actin were plotted (right panel). Results are representative of three independent experiments. Data

are presented as mean ± SD. *, $p < 0.05$; **, $p < 0.01$ (Student's t-test). **b** Confocal immunofluorescence analysis for GRP94 and LRP6 co-localization was performed using MDA-MB231 cells with depletion of CK2α and overexpression of GRP94-S306A or GRP94-S306D (left panel). The signal intensity corresponding to GRP94 and LRP6 was quantified using ImageJ software (right panel). Scale bar = 50 μm. Results are representative of three independent experiments. Data are presented as mean ± SD. ***, $p < 0.001$ (Student's t-test). **c** Interaction of LRP6 with GRP94 WT or ΔT786 was analyzed through immunoprecipitation. HEK293 cells were co-transfected with Flag-LRP6 and Myc-GRP94 WT or ΔT786 constructs. Immunoprecipitation was performed with anti-Myc antibody and immunoprecipitates were detected using indicated antibodies. For analysis, 10% of cell lysate was used as input.

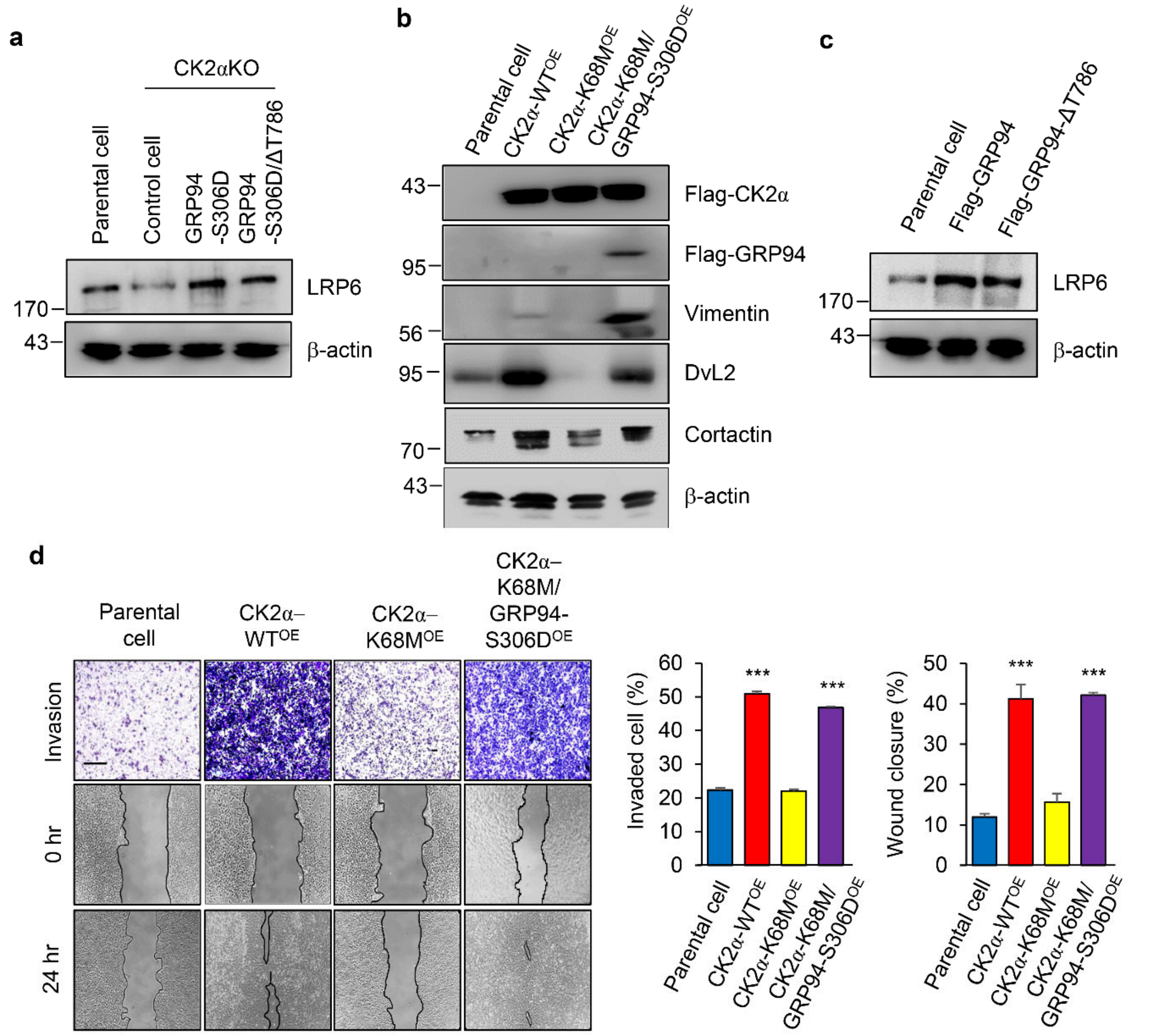

Supplementary Fig. S4 CK2α regulates LRP6 through phosphorylation of GRP94. a LRP6 protein was detected in MDA-MB231 cells with depletion of CK2α and stably expressing GRP94-S306D or S306D/ΔT786. β-actin band was used as a normalization control. **b** Wnt signaling-related markers were checked in MCF7 cells after overexpression of CK2α-WT, K68M (kinase-dead), or K68M/GRP94-S306D proteins. β-actin band was used as a normalization control. **c** LRP6 protein was detected in MCF7 cells after overexpression of GRP94-WT or ΔT786. β-actin band was used as a normalization control. **d** Transwell and wound healing assays were performed after overexpressiong CK2α-WT, K68M, or K68M/GRP94-S306D protein. The histogram shows average number of migrated and invaded cells per view (right panel). ***, $p < 0.001$ (Student's t-test). Scale bar = 200 μm.

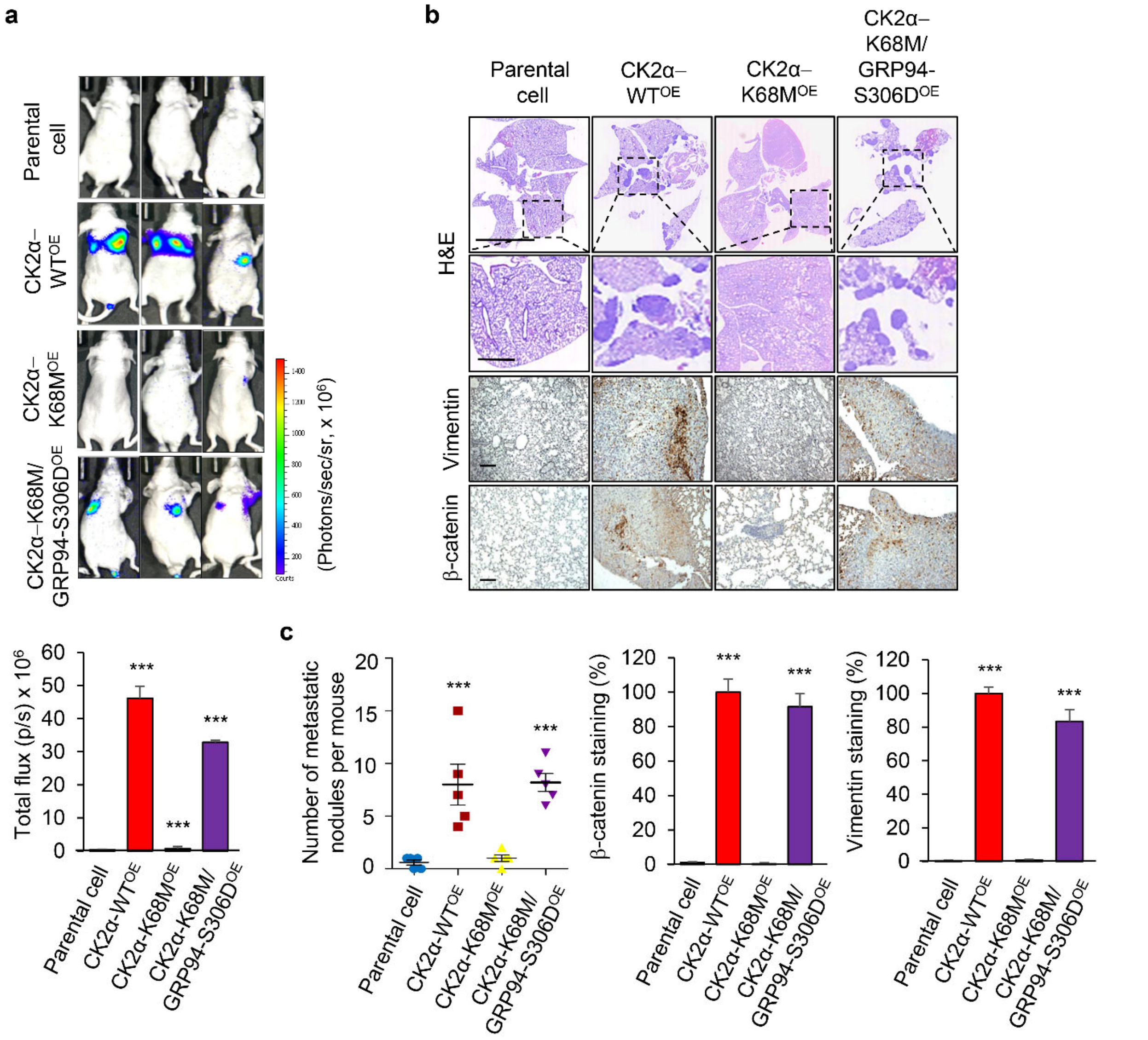

Supplementary Fig. S5 CK2α promotes metastasis of TNBC through phosphorylation of GRP94. a Representative bioluminescence images were presented after tail vein injection of indicated cells into nude mice (5 mice/group). Luciferase activity was detected using the IVIS spectrum after luciferin injection. Luminescence intensities of total flux of photons in metastatic sites of each group are presented as mean ± SD (bottom panel). ***, $p < 0.001$ (Student's t-test). **b, c** H&E (Scale bar = 200 μm (upper panel), 20 μm (bottom panel) and IHC staining of human Vimentin and β-catenin (Scale bar = 200 μm) in MCF7 cell metastatic lung tumors (**b**) and quantification of metastatic nodules and β-catenin, Vimentin expression (**c**). Scale bar = 200 μm. Data are expressed as mean ± SD. ***, $p < 0.001$ (Student's t-test).

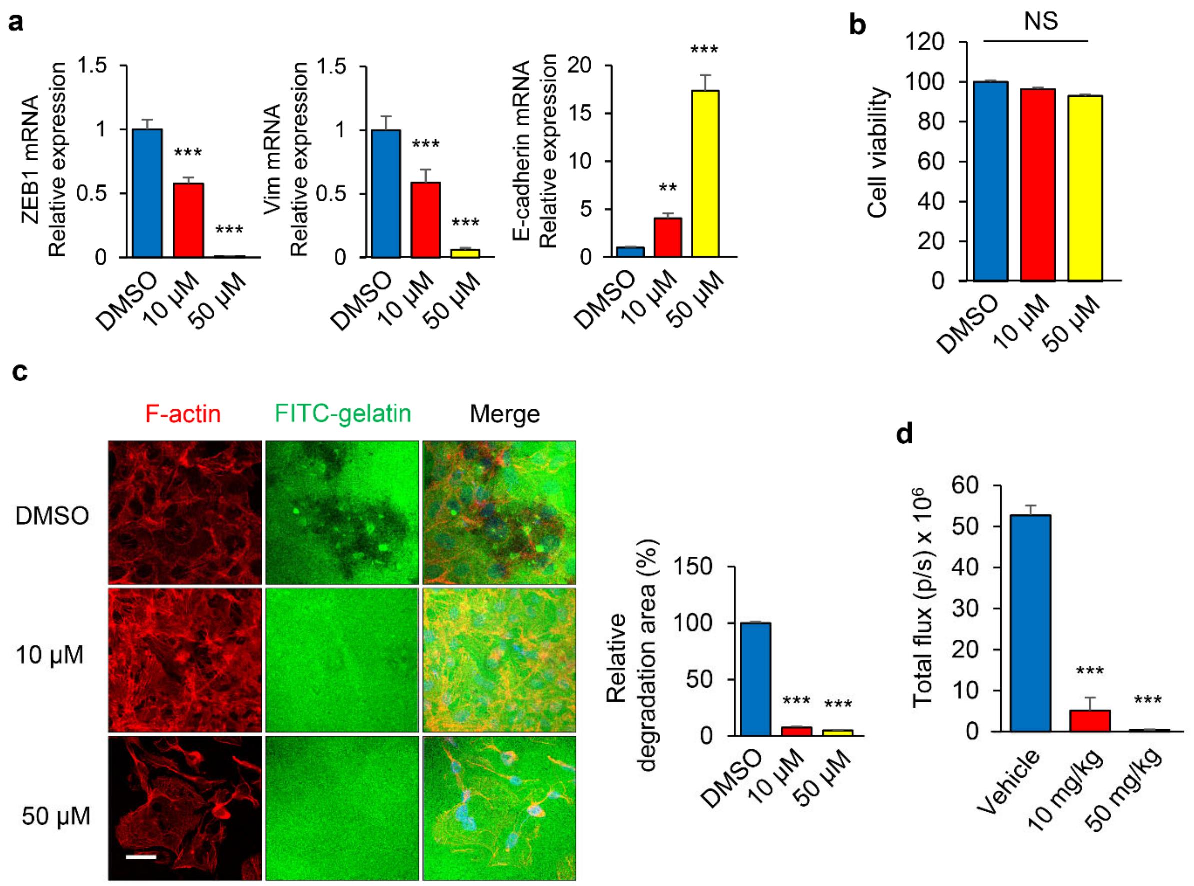

Supplementary Fig. S6 Benzamidine suppresses EMT and metastasis of breast cancer. a EMT-related markers (ZEB1, Vimentin, and E-cadherin) mRNA were validated in MDA-MB231 cells treated with benzamidine (10 μM and 50 μM) for 24 hr using qRT-PCR. Cyclophilin was used as a normalization control. **, $p < 0.01$; ***, $p < 0.001$ (Student's t-test). **b** Cell proliferation was measured in MDA-MB231 cells after treatment with benzamidine for 24 hr using cell viability assay kit. **c** FITC-gelatin degradation assay was performed after treatment with benzamidine for 24 hr. Invasion activities were measured based on the intensity of gelatin degradation. The intensity of gelatin degradation was measured using ImageJ software and compared with control cells (right panel). ***, $p < 0.001$ (Student's t-test). Scale bar = 50 μm**. d** Luciferase activity was detected using the IVIS spectrum after luciferin injection (related to Fig. 6d). Luminescence intensities of the total flux of photons in the metastatic sites in each group are presented as mean ± SD. ***, $p < 0.001$ (Student's t-test).

Supplementary Table S1. List of antibodies used in this study

Antibody	Company	Catalog number	Species	Assay	Dilution fold
GRP94	Santa Cruz Biotechnology	393402	Mouse	IB, IHC	1:1,000 for IB, 1:100 for IHC
N-cadherin	Santa Cruz Biotechnology	59987	Mouse	IB	1:1,000 for IB
Cortactin	Santa Cruz Biotechnology	11408	Rabbit	IB, IHC	1:1,000 for IB, 1:100 for IHC
β-catenin	Santa Cruz Biotechnology	7199	Rabbit	IB, IHC	1:1,000 for IB, 1:100 for IHC
CK2α	Santa Cruz Biotechnology	373894	Mouse	IB	1:1,000 for IB
p-Ser	Santa Cruz Biotechnology	81514	Mouse	IB, IP	1:1,000 for IB, 1 μg for IP
Myc	Santa Cruz Biotechnology	40	Mouse	IB, IP	1:1,000 for IB, 1 μg for IP
c-Myc	Santa Cruz Biotechnology	42	Mouse	IB	1:1,000 for IB
ZEB1	Santa Cruz Biotechnology	25388	Rabbit	IB	1:1,000 for IB
E-cadherin	Cell Signaling Technology	3195	Rabbit	IB	1:1,000 for IB
Vimentin	Cell Signaling Technology	5741	Rabbit	IB, IHC	1:1,000 for IB, 1:100 for IHC
MMP2	Cell Signaling Technology	87809	Rabbit	IB	1:1,000 for IB
LRP6	Cell Signaling Technology	2560	Rabbit	IB	1:1,000 for IB
Dvl2	Cell Signaling Technology	3224	Rabbit	IB	1:1,000 for IB
Active β-catenin	Cell Signaling Technology	8814	Rabbit	IB, IHC	1:1,000 for IB, 1:800 for IHC
CyclinD1	Cell Signaling Technology	2926	Rabbit	IB	1:2,000 for IB
Flag	Sigma-Aldrich	F3165	Mouse	IB, IP	1:3,000 for IB, 1 μg for IP
β-actin	Sigma-Aldrich	A5441	Mouse	IB	1:5,000 for IB
ZO-1	Cell Signaling Technology	5406	Rabbit	IB	1:1,000 for IB
AXIN1	Cell Signaling Technology	2087	Rabbit	IB	1:1,000 for IB

Supplementary Table S2. List of qRT-PCR primer sequence used in this study

Gene	Forward (5´ to 3´)	Reverse (5´ to 3´)
GRP94	AAGGCCTTTCACAAGTTGGC	ACGCTTCTTGGATCCAGTGTT
E-caderin	ACAGCCC`CGCCTTATGATT	TCGGAACCGCTTCCTTCA
Vimentin	AATGACCGCTTCGCCAACT	ATCTTATTCTGCTGCTCCAGGAA
c-Myc	GCCACGTCTCCACACATCAG	TCTTGGCAGCAGGATAGTCCTT
CyclinD1	AATGACCCCGCACGATTTC	TCAGGTTCAGGCCTTGCAC
LEF1	AGGAACATCCCCACACTGAC	AGGTCTTTTTGGCTCCTGCT
Cyclophilin	TGCCATCGCCAAGGAGTAG	TGCACAGACGGTCACTCAAA

Supplementary Table S3. List of primers used for mutagenesis

Primers	Sequences
CK2a-K68M-F	GAA AAA GTT GTT GTT ATG ATT CTC AAG CCA GTA
CK2a-K68M-R	TAC TGG CTT GAG AAT CAT AAC AAC AAC TTT TTC
GRP94-S306A-F	GAG AAA GAA GAA GCT GAT GAT GAA GCT
GRP94-S306A-R	AGC TTC ATC ATC AGC TTC TTC TTT CTC
GRP94-S306D-F	GAG AAA GAA GAA GAT GAT GAT GAA GCT
GRP94-S306D-R	AGC TTC ATC ATC ATC TTC TTC TTT CTC
GRP94-wildtype-F	GATC GAATTC A ATG AGG GCC CTG TGG GTG CTG GGC CTC TGC
GRP94-ΔT786-R	GAT CCT CGA GCA CAT CCA TTT CTT CAT CTT C

Supplementary Table S4. Predictive analysis of GRP94 phosphorylating kinases

Human GRP94 S306 (EKEESDDEA) phosphorylating kinases

Kinase	NetPhos	ScanSite	Phospho Motif finder	GPS
CK2	O	O	O	O
AMPK				O
PRKAA1				O
AGC				O
CMGC				O

Human GRP94 T786 (MDVGTDEEE) phosphorylating kinases

Kinase	NetPhos	ScanSite	Phospho Motif finder	GPS
CK2	O	O	O	O
CK1	O			O
AGC				O
PKA				O
CMGC				O
