# Supplementary material for: CK2α-mediated phosphorylation of GRP94 facilitates the metastatic cascade in triple-negative breast cancer
Source: Cell Death Discov. 2024 Apr 22;10:185. doi: 10.1038/s41420-024-01956-x (PMC11035675; doi:10.1038/s41420-024-01956-x)

Fig 1. a

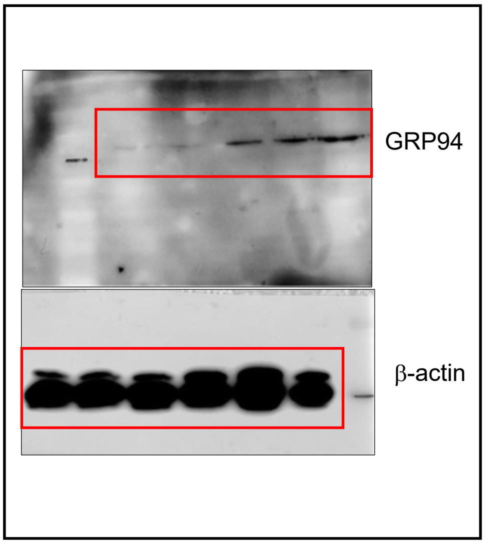
GRP94
β-actin

Fig 1. d

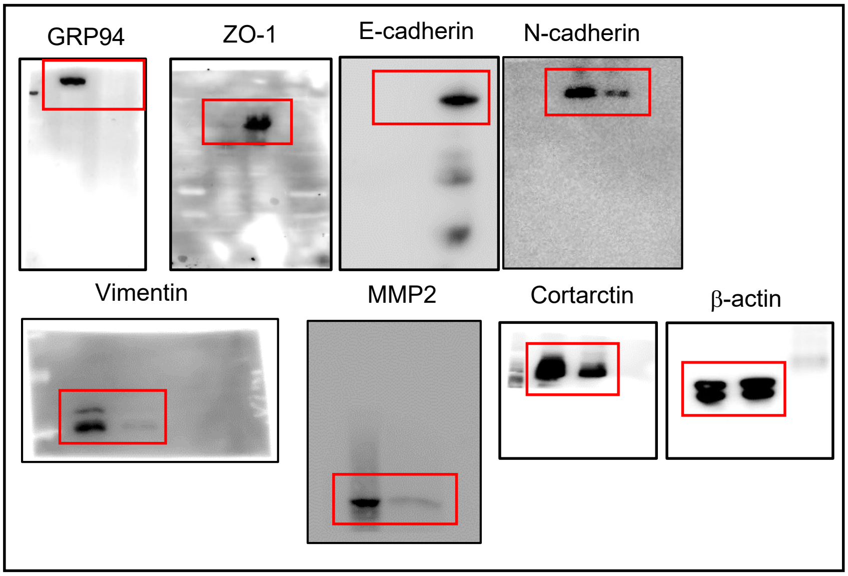
GRP94
ZO-1
E-cadherin
N-cadherin
Vimentin
MMP2
Cortarctin
β-actin

Fig 2. c

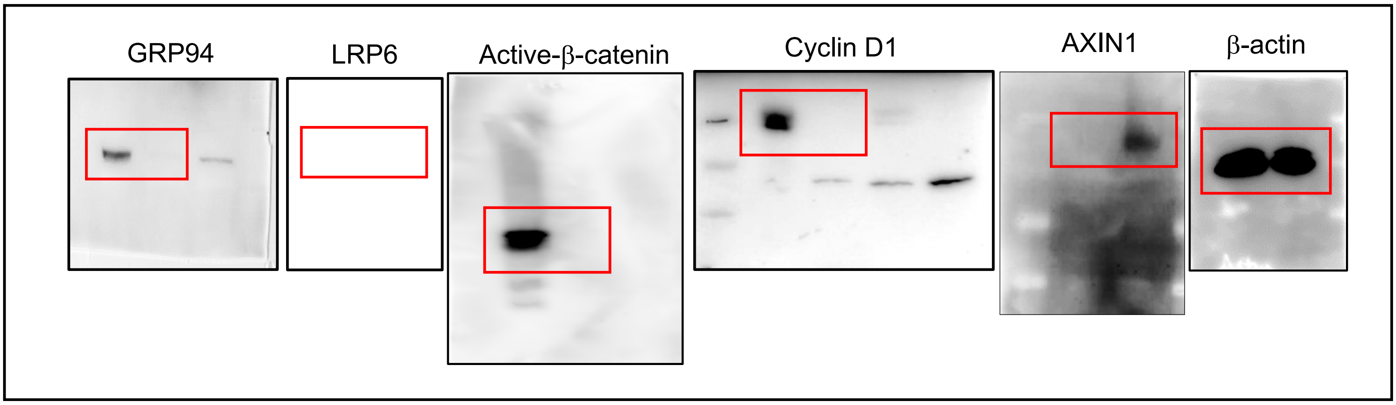

Fig 2. d

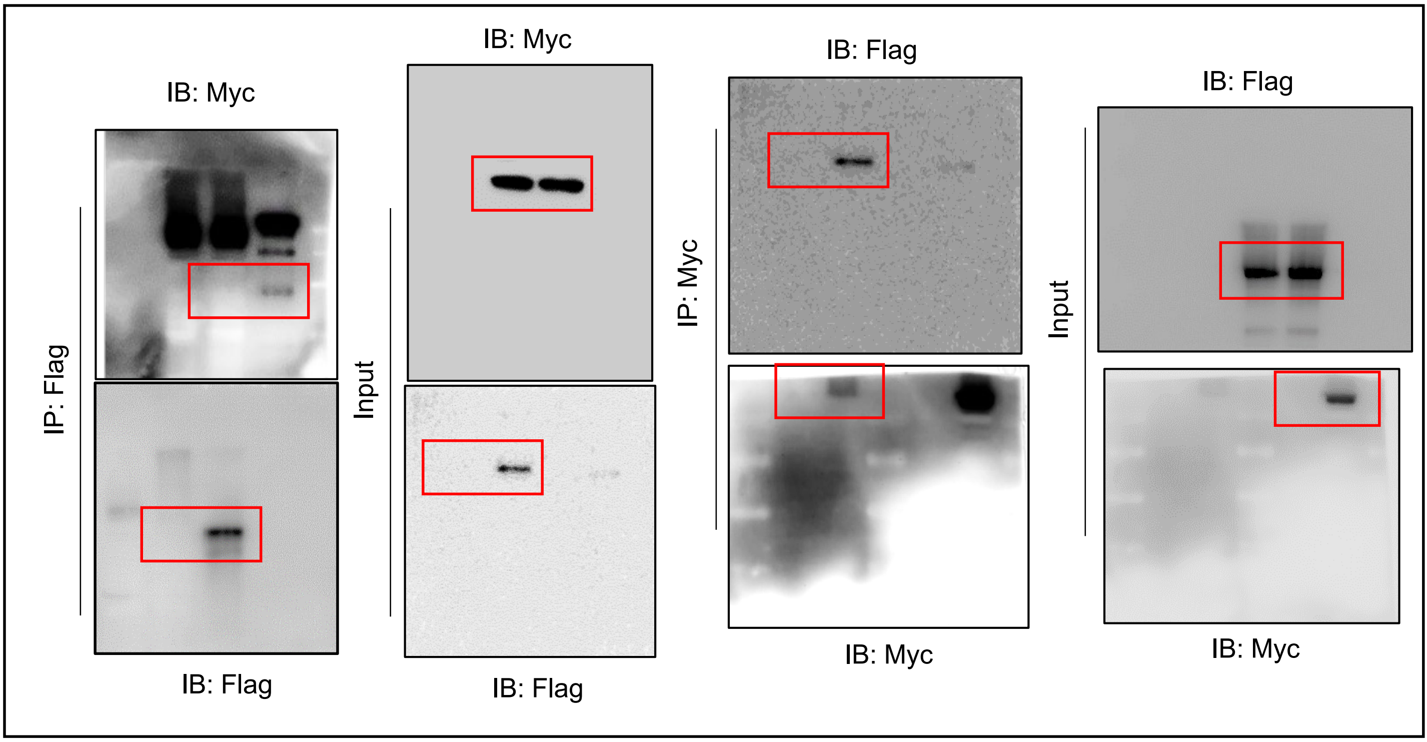

Fig 2. e

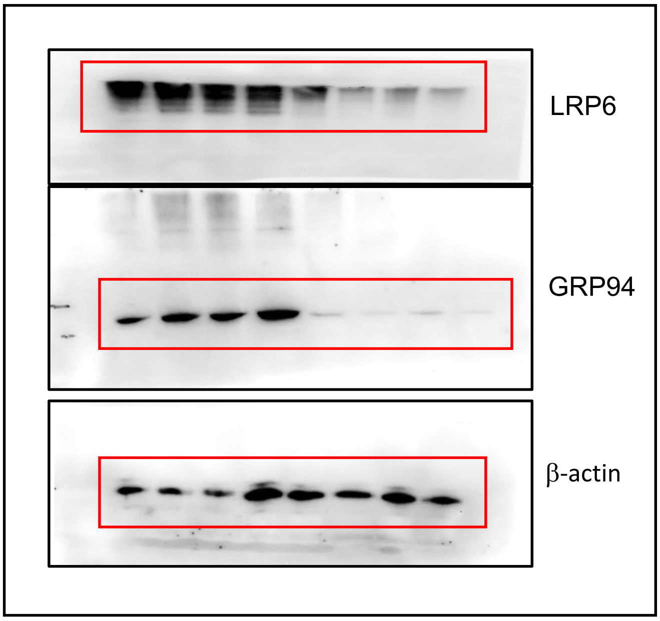

Fig 3. a

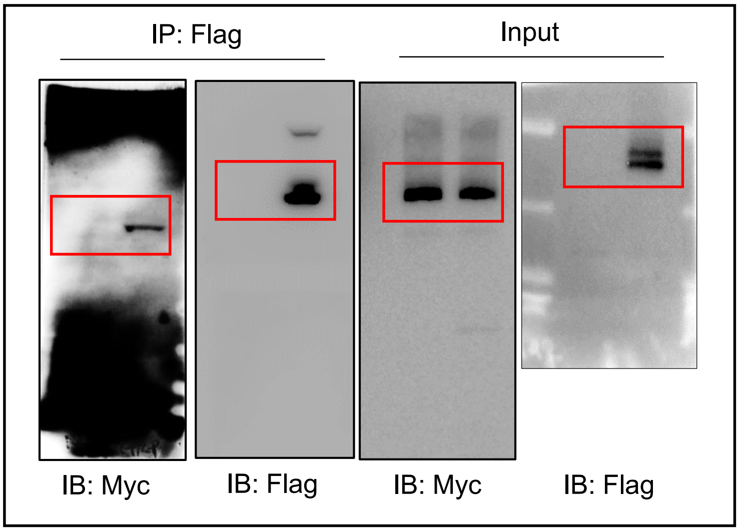

Fig 3. b

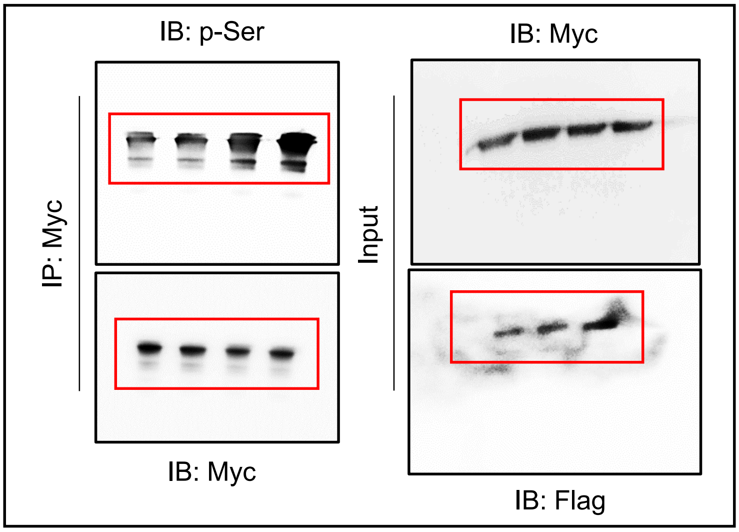

Fig 3. c

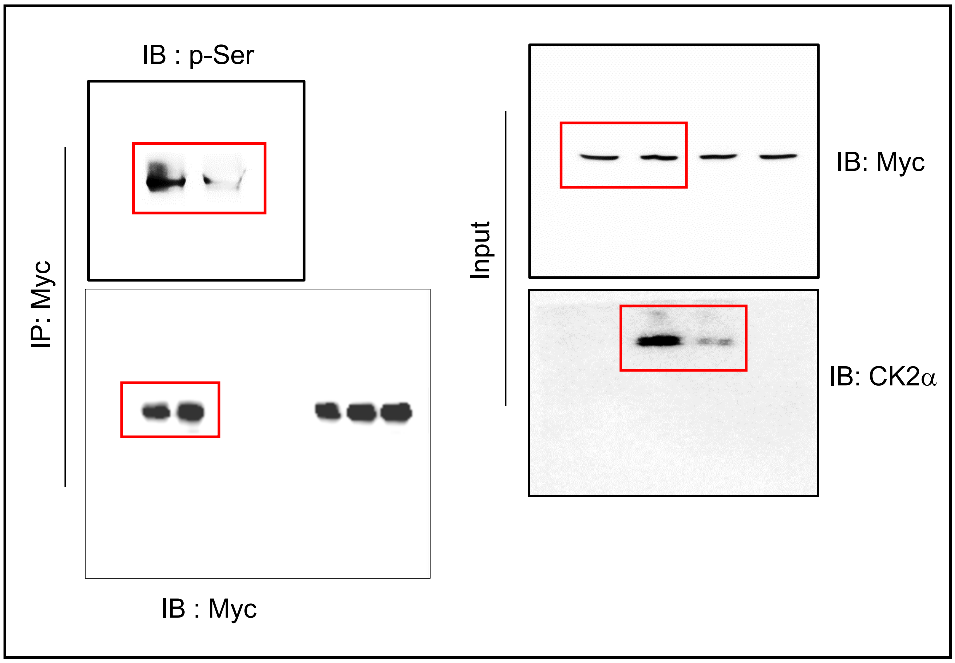

Fig 3. d

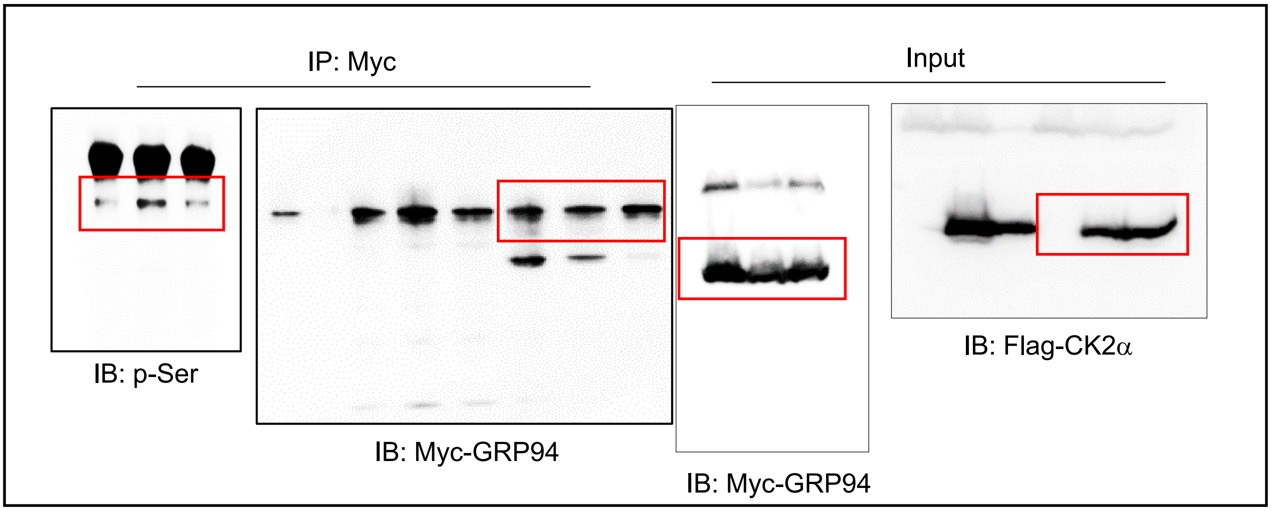

Fig 3. e

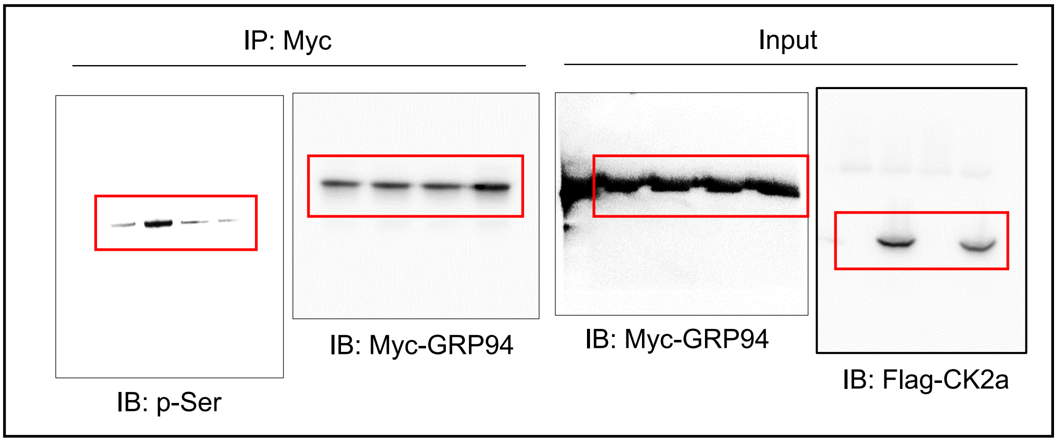

Fig 3. f

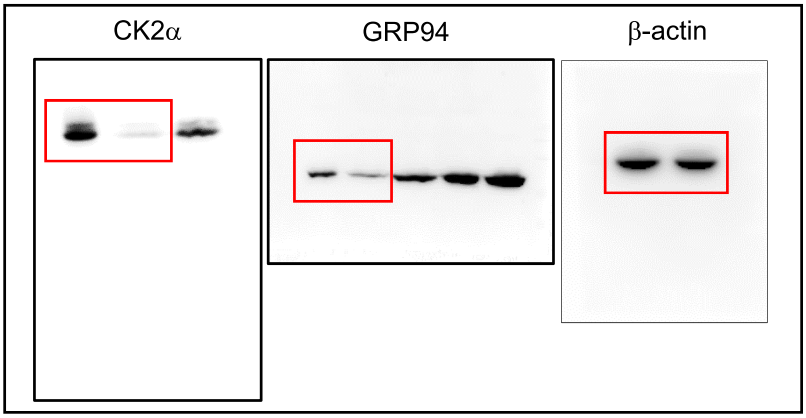

Fig 3. g

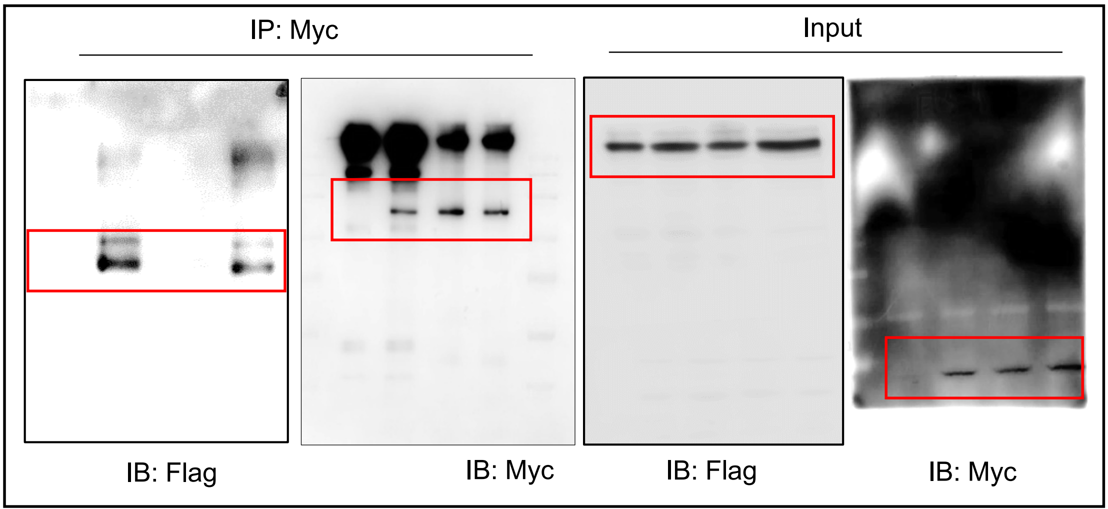

Fig 4. a

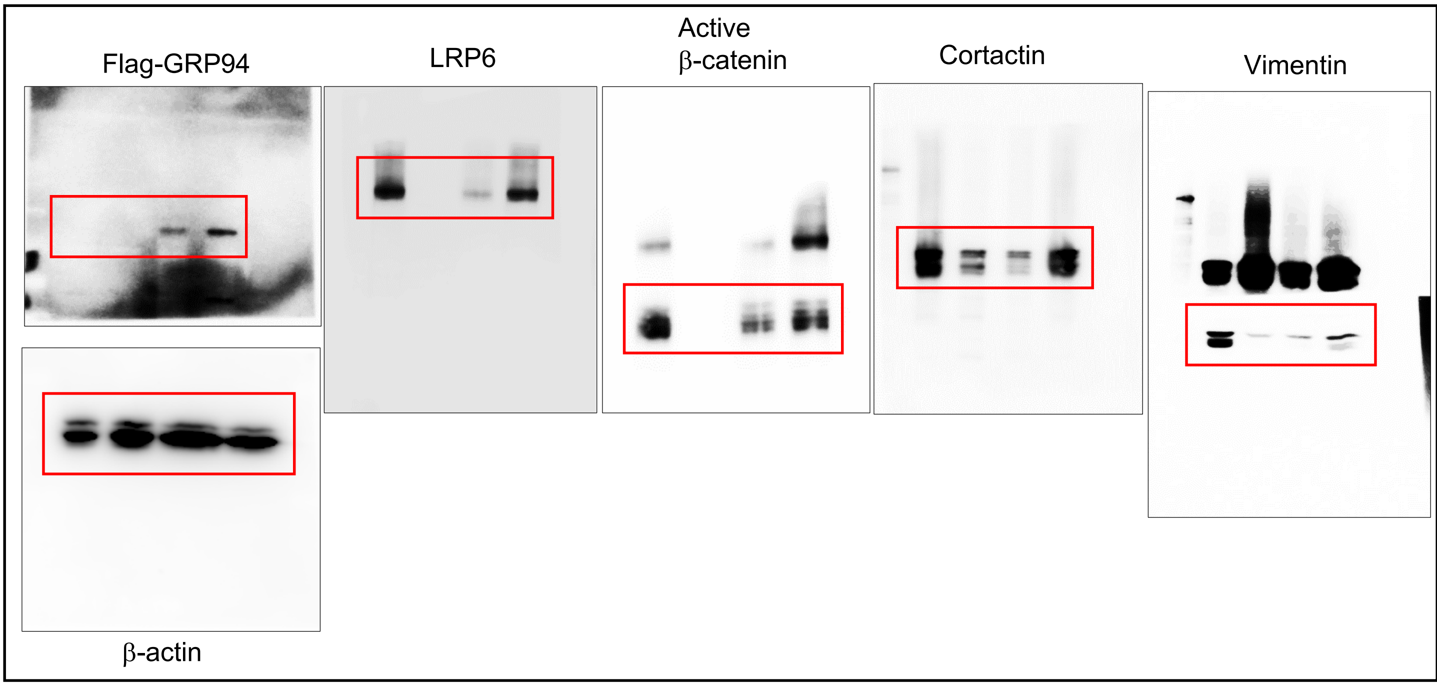

Fig 5. d

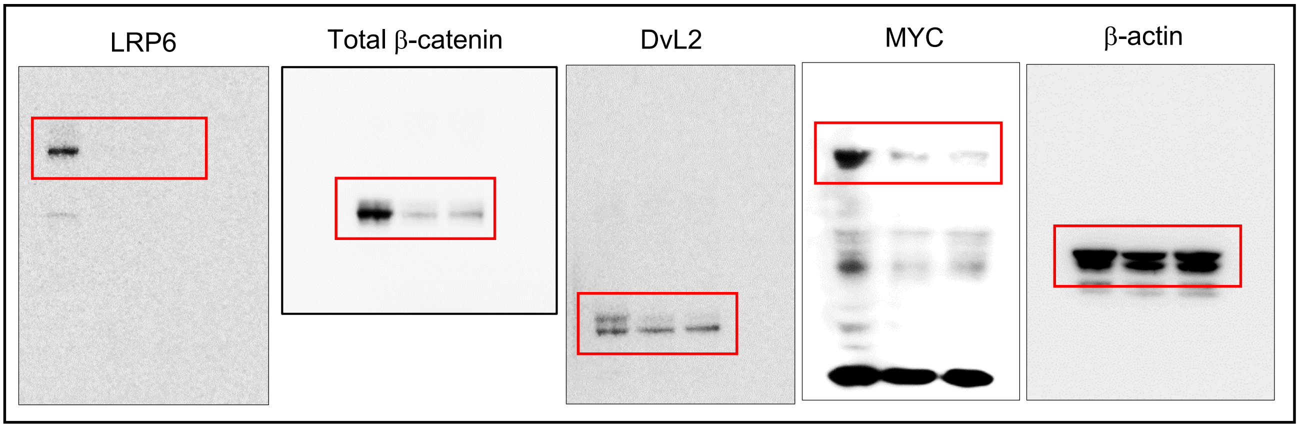

Fig 5. e

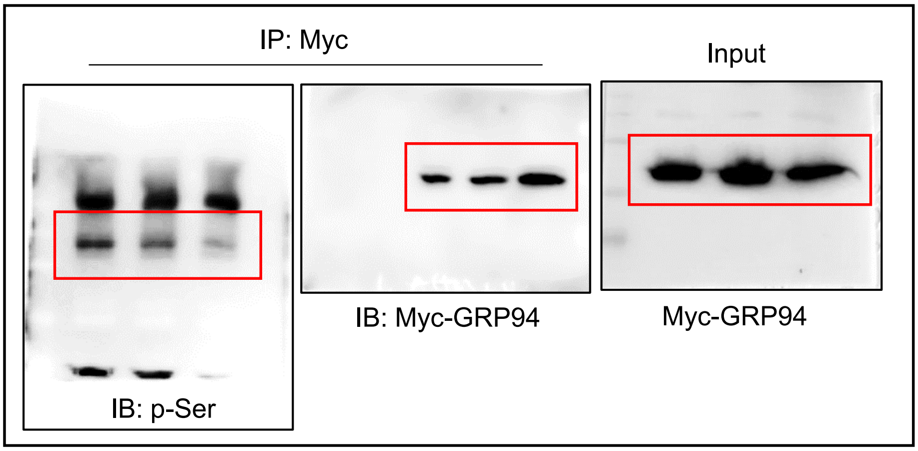

Fig 5. f

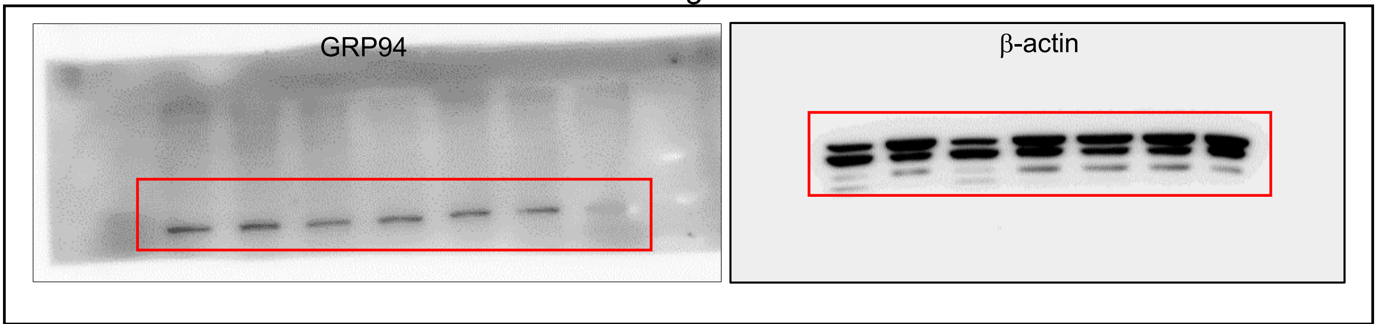

Fig 6. a

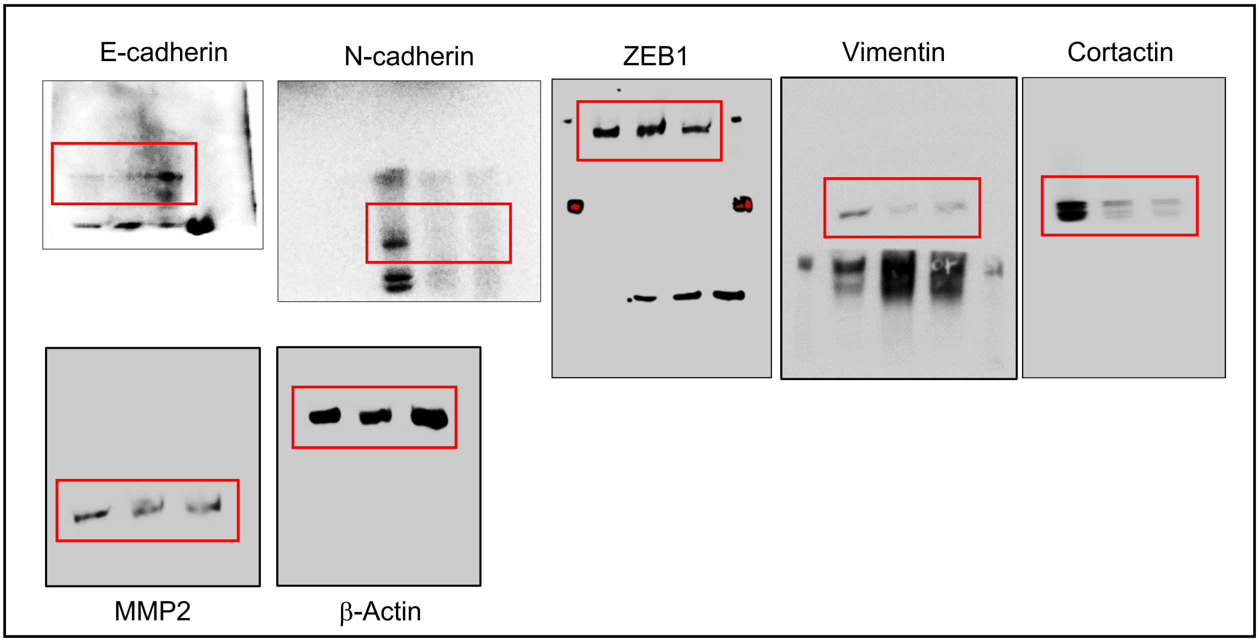

Sup Fig 1. b

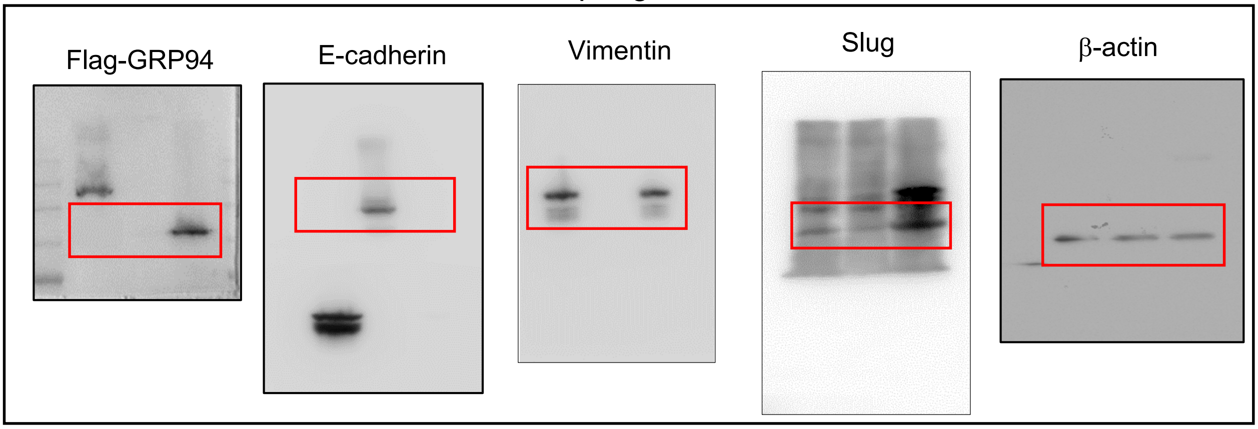

Sup Fig 1. d

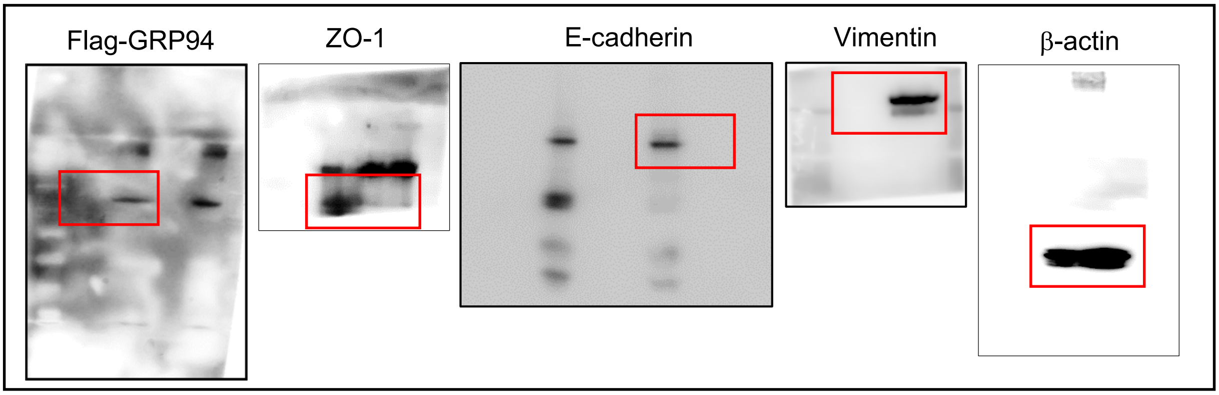

Sup Fig 2. b

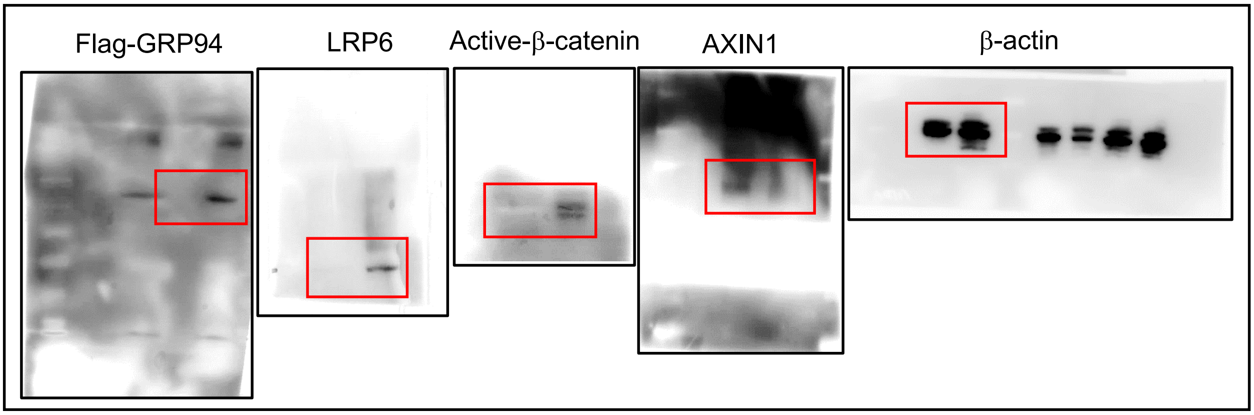

Fig sup. 3a

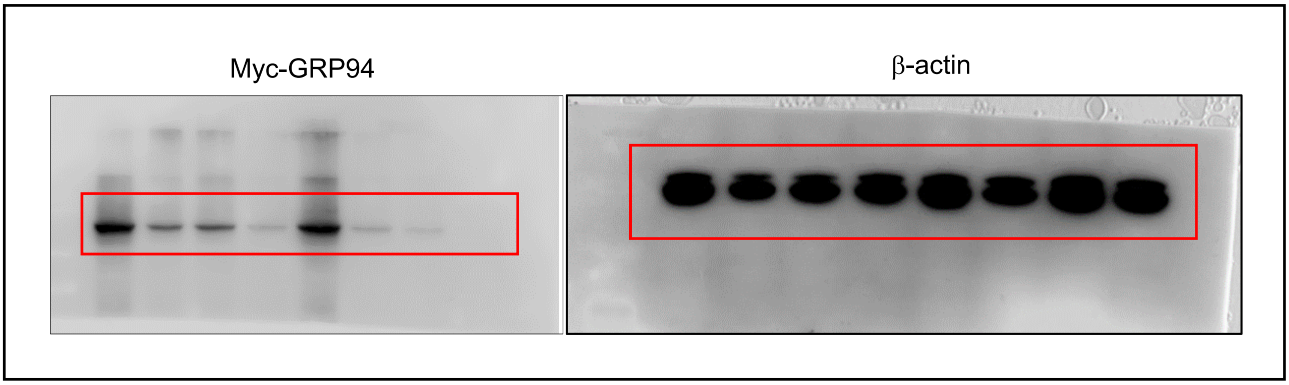

Fig sup. 3c

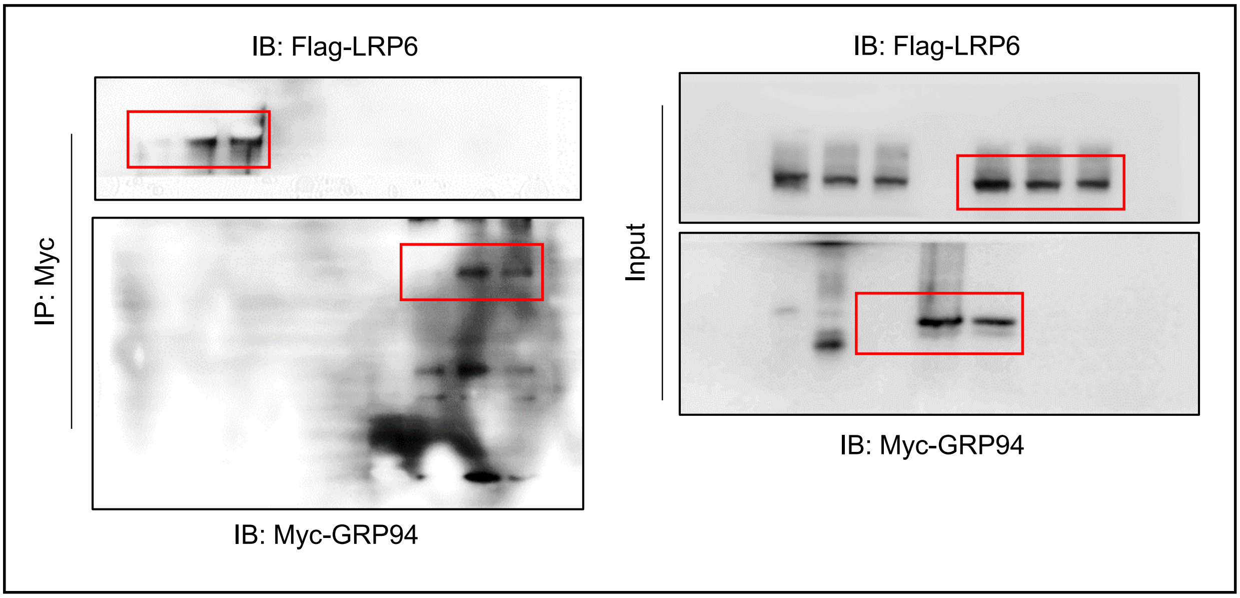

Fig sup. 4a

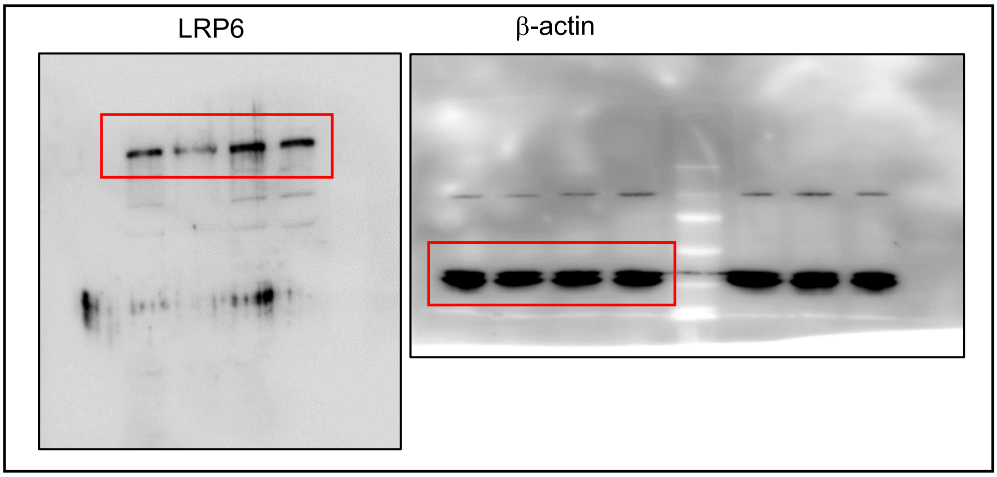

Fig sup. 4b

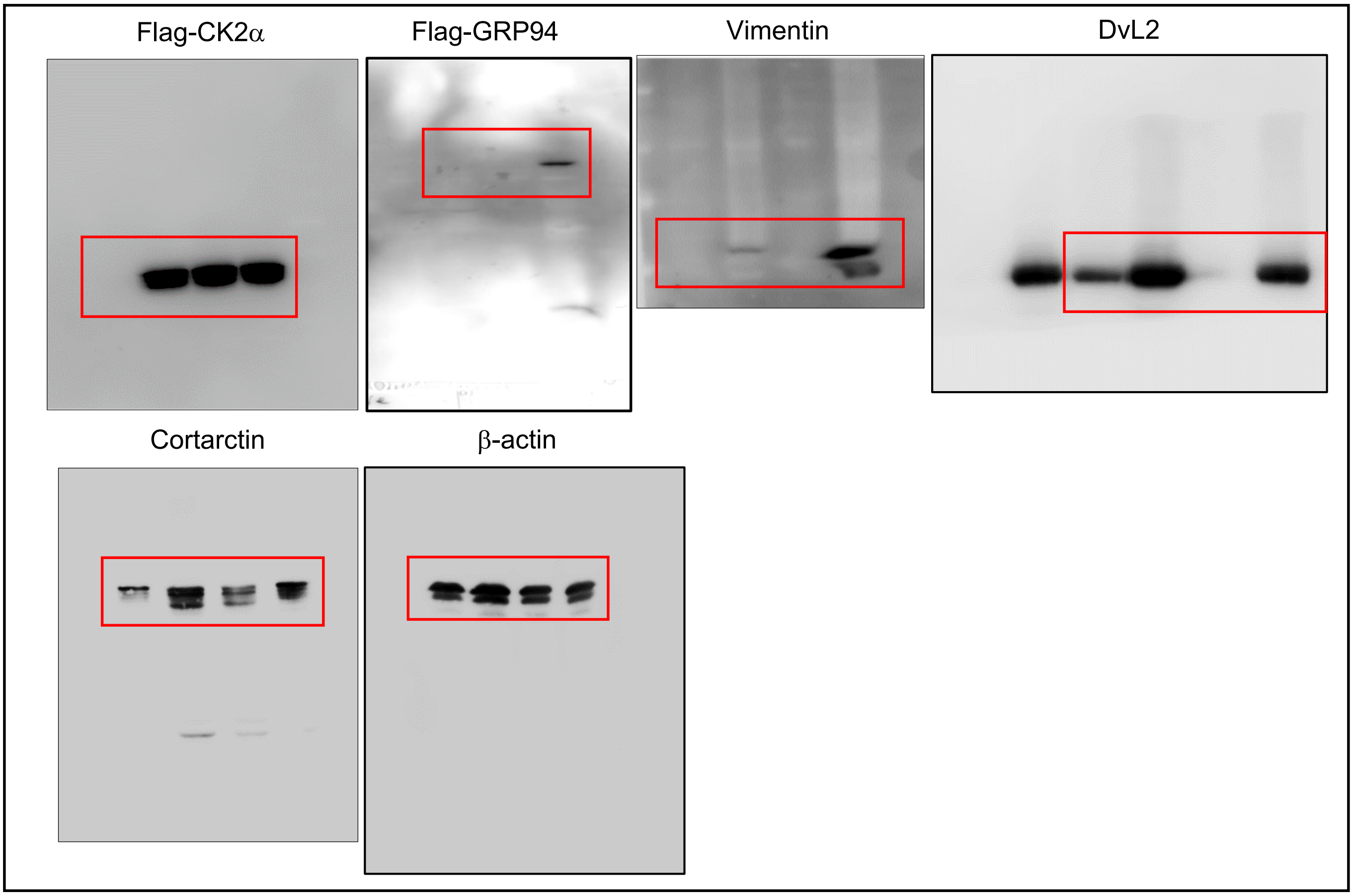

Fig sup. 4c

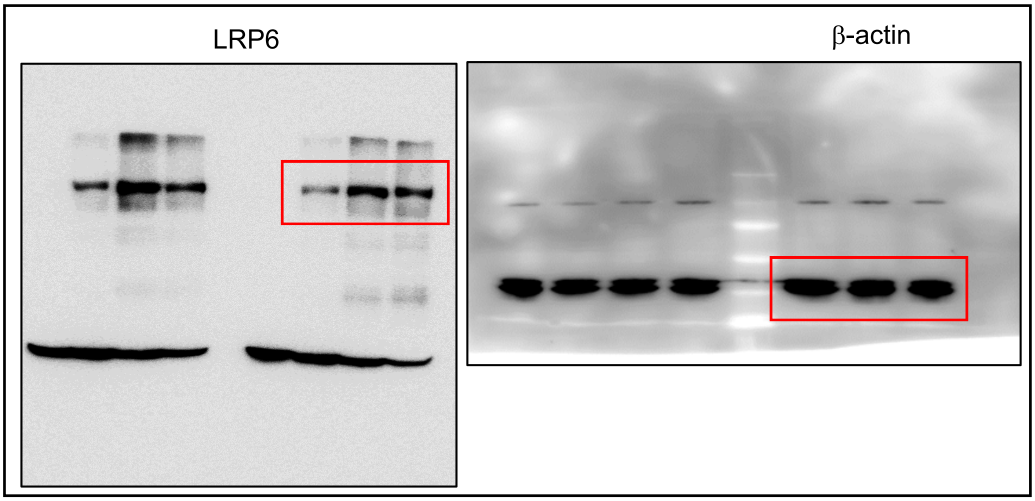

Supplement: Supplementary file 2 — Original Data File [file 41420_2024_1956_MOESM2_ESM.pdf]
